# Finite Element Analysis of Fiber-Reinforced Pneumatic Soft Actuators: A Hybrid Analytical–Numerical Framework

**DOI:** 10.3390/ma19173631

**Published:** 2026-08-26

**Authors:** Ruibing Fan, Guowei Shao, Jianhua Tang, Yao Wang, Pengyu Xu

**Affiliations:** 1 School of Intelligent Manufacturing, Changzhou Vocational Institute of Textile and Garment, Changzhou 213164, China; rbfan@cztgi.edu.cn (R.F.); gwshao@cztgi.edu.cn (G.S.); jhtang@cztgi.edu.cn (J.T.); jdywang@cztgi.edu.cn (Y.W.); 2 The Jiangsu Research Center of Intelligent Manufacturing Technology for Carbon Fiber and Advanced Material, Changzhou 213164, China; 3 Engineering Research Center of Artificial Intelligence for Textile Industry Ministry of Education, Institute of Artificial Intelligence, Donghua University, Shanghai 201620, China; 4 School of Material Science and Engineering, Zhengzhou University, Zhengzhou 450001, China

**Keywords:** soft actuator, fiber reinforcement, finite element analysis, parametric design, stress distribution, pneumatic actuation

## Abstract

Pneumatic soft actuators have been drawing considerable attention in the field of soft robotics, thanks to their inherent flexibility, high power density, and safe interaction. However, the strong, intricate coupling between the material’s hyperelastic behavior and the reinforcement of anisotropic fibers creates significant challenges for both analytical modeling and numerical characterization of these actuators. In this paper, we design and fabricate a fiber-reinforced pneumatic soft actuator using Ecoflex 00-30 silicone rubber as the base material and helically wound fibers as the reinforcing layer. We set up a theoretical framework that combines the Neo-Hookean model for isotropic silicone rubber with a strain energy-based formulation for anisotropic wound fibers. This framework describes how the actuator is stretched, expanded, twisted, and bent. Finite element simulations are then carried out, focusing on three key design parameters: winding fiber density (three levels: high, medium, low), air cavity offset distance from the central axis (1, 2, 3, and 4 mm), and air cavity cross-sectional geometry (cube vs. cylindrical). The simulations reveal that a higher winding fiber density promotes more uniform stress distribution across both the strain and confinement layers. In contrast, a low fiber density can lead to local bulging and large stress variations, which ultimately compromises the bending performance. The offset distance of the air cavity from the neutral axis is directly linked to the bending curvature: a larger offset produces greater air cavity deformation and higher actuation efficiency. Furthermore, the cuboid air cavity yields a larger bending angle (experimentally validated up to 90° at 0.045 MPa) and better efficiency, while the cylindrical air cavity distributes stress more evenly across the outer surface of the strain layer and reduces stress concentration at the edges. These findings provide useful quantitative guidance for optimizing the structure of fiber-reinforced soft actuators and establish a framework for hybrid analytical–numerical prediction of their mechanical behavior.

## 1. Introduction

Soft robots have rapidly developed as an exciting interdisciplinary field. It draws inspiration from mechanical engineering, materials science, and textile engineering to build machines [1]. These machines can safely switch between environments and adapt to unstructured situations. Unlike conventional robots that are built from rigid materials, soft robots are made from flexible materials that allow them to deform continuously, move freely, and withstand shocks. This suggests that it is particularly well-suited for applications like medical rehabilitation, gripping agricultural tools, underwater handling, and interaction. The heart of a soft robot system is the soft actuator, which is primarily in charge of producing movement and force. We have looked at several ways that soft actuators work, including shape memory alloys, electroactive polymers, a tension-driving mechanism, and a pneumatic system. Among these, shape memory alloy (SMA)-based actuators offer high force density and a simple structural format but typically suffer from slow response due to thermal cycling, limited actuation frequency, and low energy efficiency, which restricts their use in applications requiring rapid or continuous motion [2]. Electroactive polymer (EAP) actuators, including dielectric elastomers and ionic polymer–metal composites, provide fast response, lightweight construction, and large actuation strains; however, their output force is generally low, and they require high driving voltages (typically several kV for dielectric elastomers) or an electrolyte environment, which complicates practical implementation [3]. Tendon- or cable-driven actuators enable precise position control through external motors and are widely used in wearable and rehabilitative devices, but their reliance on rigid transmission components compromises the inherent compliance and adaptability that define soft robotics [4]. In comparison, pneumatic soft actuators offer a favorable combination of fast response, high power density, large actuation force, inherent compliance, and safe operation under low driving pressures (typically 0–0.2 MPa), making them particularly attractive for applications in medical rehabilitation, agricultural grasping, and human–machine interaction. These characteristics motivate the choice of pneumatic actuation as the driving method in the present study. This is due to their speed, strength, ability to carry heavy loads, and working under safe conditions [5]. The elastomer matrix, typically made of silica gel rubber, provides the flexibility for deformation while serving as the foundation for converting mechanical energy into aerodynamic energy.

While it has many advantages, the bearing capacity and directional control of pure elastic pneumatic actuators are also limited. To overcome these shortcomings, fiber reinforcement is a common strategy [6,7,8]. By winding high-modulus fibers around the actuator’s flexible body, radial expansion is effectively limited, leading to improved axial elongation, structural strength, and directional control of deformation. The effective angle, density, and fiber arrangement all play a key role in shaping the performance of the final execution. The combination of a hyper-elastic isothermal matrix with anisotropic fiber reinforcement creates a composite structure whose mechanical behavior is dictated by the interaction between the matrix and fiber components. Previous research has explored various aspects of fiber-reinforced soft actuators, including how the fiber angle affects actuator elongation and the creation of analytical models for predicting actuator performance [9]. But it is not obvious how structural parameters like fiber density in rotation, cavity eccentricity, and cavity cross-sectional shape impact stress distribution and overall deformation in bending. Modeling fiber-reinforced soft actuators is a significant theoretical challenge. The silicone rubber matrix is hyperelastic and nearly incompressible, exhibiting nonlinear behavior. This prevents the model from being used in its usual way [10]. The Neo-Hookean constitutive model, which is based on the statistical thermodynamics of polymer network, has been widely applied to study the mechanical response of silicone elastomers under finite deformation.

The model uses deformation energy density as a function of the gradient deformation tensor invariants, which represent the key features of material hyperelastic deformation, such as volume conservation and nonlinear elasticity. On the other hand, anisotropic wound fibers require different formulas for their deformation energy, taking into account the orientation of the fibers, their axial stiffness, and how they affect the surrounding matrix material. In addition, the coupling between the compressed air in the cavity and the deformable elastomer wall adds another layer of complexity to the overall mechanical analysis. It is extremely difficult to describe the stress and deformation field in the actuator when it is running. This is due to both the opacity of silicone rubber and the fact that air cavities are built in. These factors illustrate why a combined method of analysis and numerical calculation is needed to reveal the inner mechanical state of the actuator under different design settings [11].

Finite element analysis (FEA) has demonstrated its effectiveness as a tool for studying the mechanical behavior of soft actuator composites. By coupling solid mechanics and fluid mechanics formulations within a unified numerical framework, we can simulate the interaction between compressed air and hyperelastic silicone rubber. The material parameters obtained from experiments can then be calibrated through standard numerical optimization procedures. This numerical approach provides a systematic means to analyze how the stress distribution and strain response of actuators vary across different design parameters.

Previous studies on fiber-reinforced soft actuators have largely focused on the effect of a single design parameter. The influence of fiber winding angles on axial elongation has been investigated analytically and experimentally, with recent work by Shabani et al. further examining how tilted-helical fiber orientation affects coiling deformation combining bending and torsion [12]. Winding density and its relationship to bending performance have been studied in detail by Tan et al., who showed through systematic FE simulation that increasing fiber turn count from 9 to 100 progressively reduces radial bulging and increases bending angle in both single-helix and double-helix configurations [13]. Esmalipour et al. developed a 3D FE model of Kevlar fiber-reinforced silicone actuators and identified optimal fiber pitch (1.5 mm) and layer thickness (1 mm) for maximizing bending angle, using hyperelastic material parameters calibrated by ASTM D412 tensile testing [14]. Regarding cavity design, the effects of cavity cross-sectional shape on actuation performance have been studied for various polygonal geometries [15], while Dong et al. developed an analytical framework for flexible actuators with eccentric chambers [16]. Parametric optimization of core radius, actuator length, and wall thickness for linear fiber-reinforced actuators has also been reported. However, a systematic comparative analysis of how fiber winding density, air cavity eccentricity, and cavity cross-sectional geometry jointly influence the internal stress distribution and bending deformation of fiber-reinforced actuators has not been reported. Furthermore, while analytical models have been developed for fiber-reinforced actuators undergoing extension and inflation, a unified framework that simultaneously captures extension, expansion, twist, and bending—and explicitly maps the analytical formulation to a finite element implementation with experimental validation—remains largely unexplored.

This study aims to bridge the gap by creating a detailed analysis model of fiber-reinforced pneumatic soft actuators, then systematically investigating their stress distribution and deformation characteristics through finite element simulation. The remainder of this paper is organized as follows. Section 2 describes the design rationale, actuation principle, and fabrication procedure of the fiber-reinforced pneumatic soft actuator. Section 3 presents the analytical constitutive framework, including the Neo-Hookean model for the isotropic silicone rubber matrix and the strain energy formulation for the anisotropic wound fibers, and derives the governing equations for extension, expansion, twist, and bending deformations. Section 4 details the finite element simulation setup and systematically evaluates the influence of three key design parameters—winding fiber density, air cavity offset distance, and cavity cross-sectional geometry—on the stress distribution and bending performance of the actuator. Finally, Section 5 summarizes the main findings, discusses the limitations of the current model, and outlines directions for future work.

## 2. Actuator Design and Fabrication

### 2.1. Design Rationale and Actuation Principle

Unlike most other actuation methods, pneumatic systems stand out for their fast response, high power, and ability to handle large loads [17]. The material we used was silicone rubber, which is flexible enough to withstand large bending deformations while keeping the air pressure at a safe level [18]. Consequently, pneumatic soft actuators offer two key advantages: they can generate motion effectively and fail in a compliant, safe manner.

**Figure 1:**
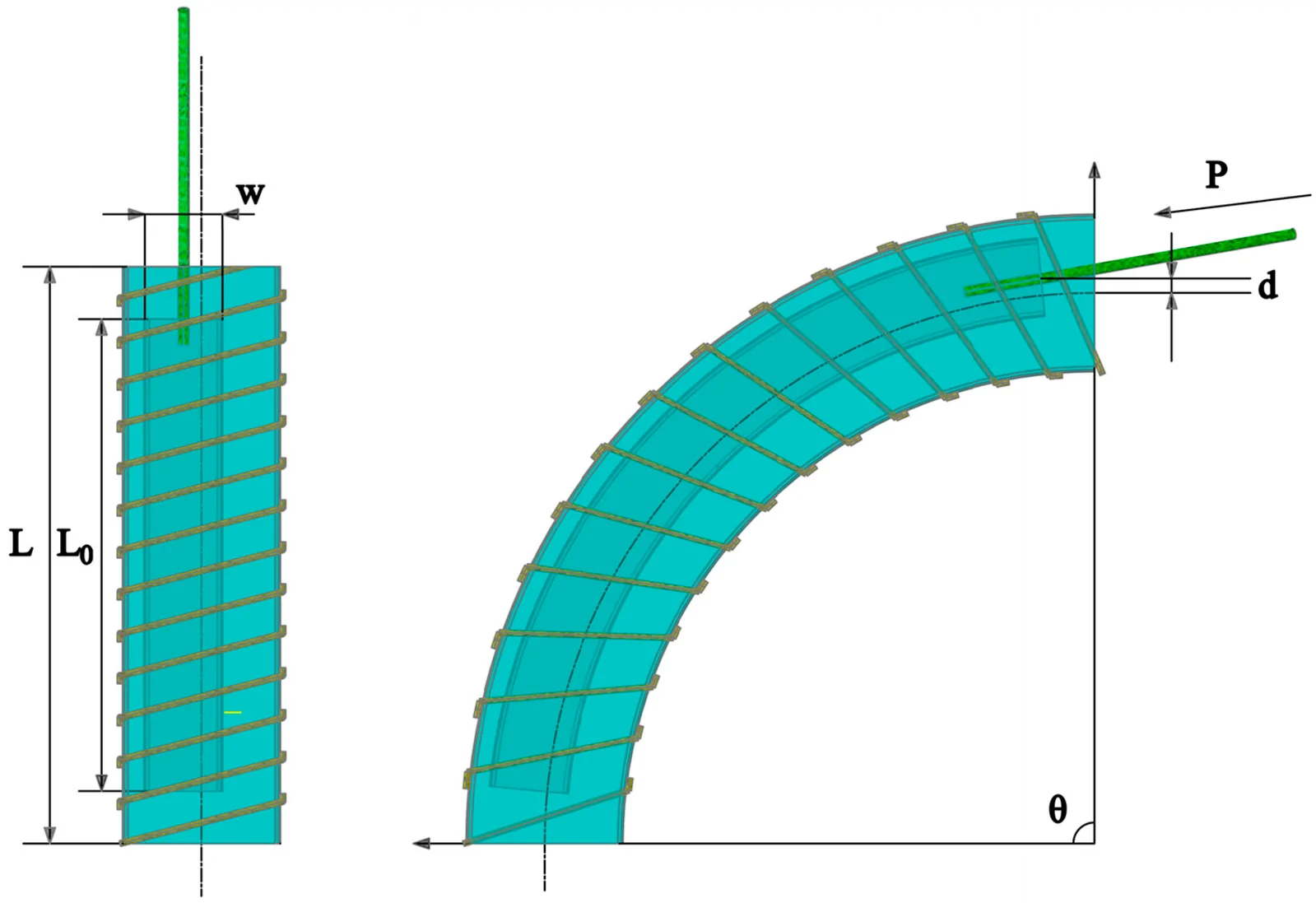
Fiber-reinforced soft actuator with offset central axis of the air chamber.

The pneumatic soft actuator consists of a body that is both hyperelastic and contains an embedded cavity. Figure 1 shows the working principle of the device. When compressed air *P* is applied, the cavity expands, creating a noticeable movement. This mechanism sets up a direct connection between internal pressure and movement.

Two geometric parameters are particularly important: the total length of the actuator (L) and the length of the cavity (L_0_). The internal cavity has a width W, which, together with L_0_, defines the cavity volume and influences the expansion characteristics. A key design variable is the offset distance d, defined as the distance between the centerline of the air cavity and the neutral axis of the actuator body; this eccentricity induces asymmetric deformation upon pressurization. During actuation, compressed air is supplied at pressure *P*, and the resulting bending deformation is quantified by the bending angle *θ*. These parameters form the basis of the parametric study presented in Section 4. The mechanical properties of the actuator are based on its cavity shape, the type of elastomer used, and the thickness of its walls. By systematically changing these parameters, the actuator’s motion can be adjusted without altering its fundamental operating principle.

A key design feature is that the cavity is located off-center relative to the axis of the actuator (see Figure 1). When pressure is applied, the soft part of the wall expands first, causing it to deform asymmetrically. The local flexibility of this design is primarily determined by the distribution of shell thickness.

### 2.2. Fabrication Procedure

Figure 1 illustrates the actuator geometry and the eccentric cavity responsible for asymmetric deformation. The cavity needs to be highly expandable as it is the fundamental structural element of the soft actuator. Silicone rubber is well suited for the air cavity because it undergoes large reversible deformations under pressure and fully recovers upon depressurization [19,20]. The actuator’s bending response can be tuned by modifying the cavity geometry—specifically the wall thickness, cross-sectional shape, and cavity length—each of which measurably affects the final mechanical behavior. Figure 1 also shows that the air cavity is the main functional element, putting strict demands on the material stretchability. Silicone elastomers conform to this requirement well, deforming significantly under pressure and quickly recovering their original shape once the pressure is gone. From a design point of view, the actuator response can be programmed by modifying the cavity geometry, such as wall thickness, cross-sectional design, and cavity length. These geometric variables all have a measurable impact on the final mechanical properties.

**Figure 2:**
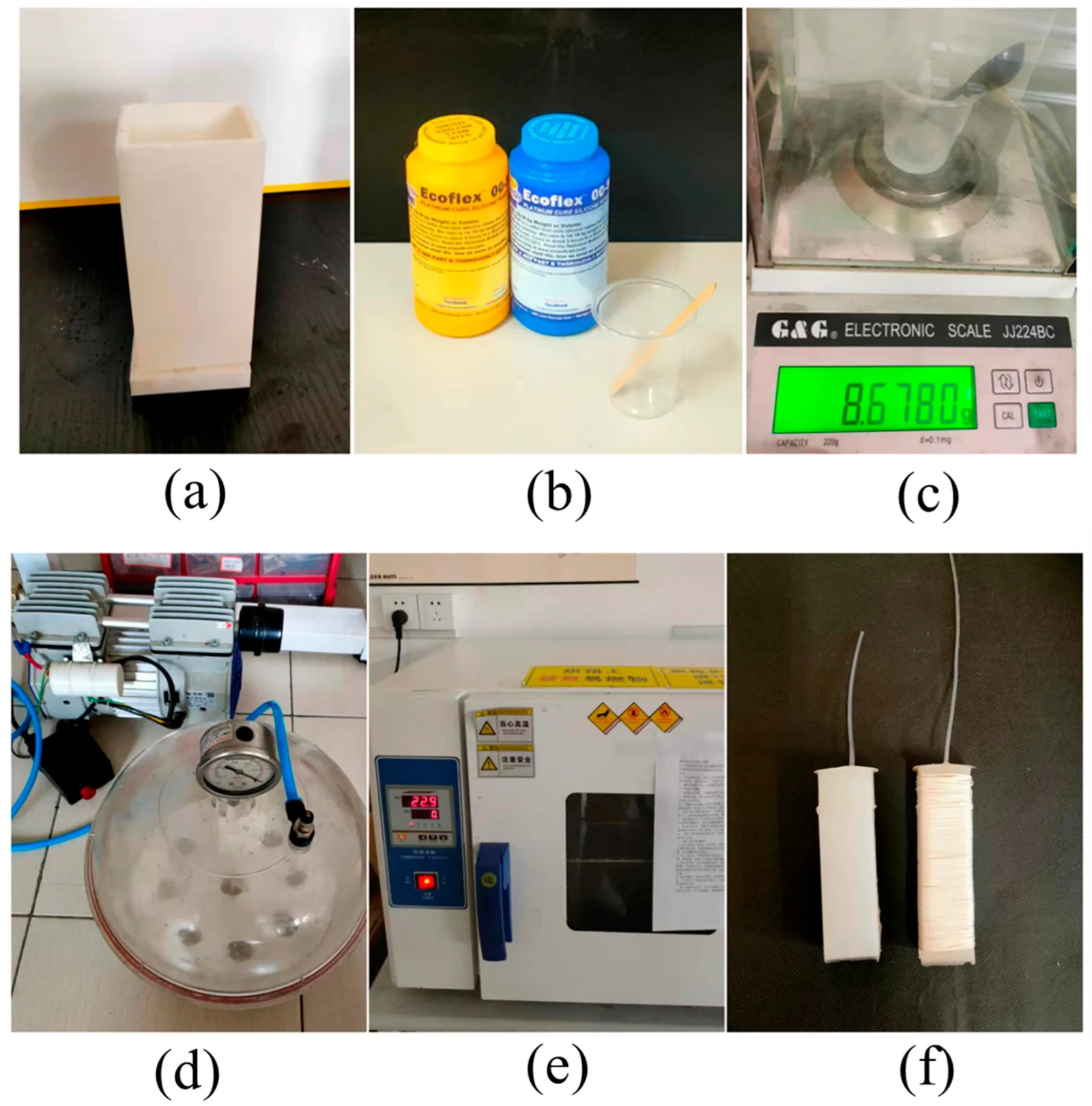
Manufacturing process of soft actuator: (**a**) 3D-printed mold assembly; (**b**–**d**) preparation of the silicone mixture (weighing, mixing, and degassing); (**e**) casting and curing; (**f**) demolding and sealing.

Soft actuators are crafted in a series of molding and assembly steps, as detailed below and illustrated in Figure 2:

Step 1—Mold assembly (see Figure 2). The three-dimensional printed mold parts, comprising the base, inner core, and side cover, are assembled to match the desired cavity shape. The three-dimensionally printed mold—comprising the base, inner core, and side cover—is assembled and sealed with hot-melt adhesive to prevent leakage of the liquid silicone precursor.

Step 2—Figure 2b–d describe the preparation of silicone. The measurement of the parts of Ecoflex 00-30 (Smooth-On, Inc., Macungie, PA, USA) silicone elastomer was performed by an electronic balance, (Changshu Shuangjie Testing Instrument Factory, Changshu, China) followed by thorough mixing in a plastic cup at a 1:1 weight ratio. The mixture is vacuumed to remove any air bubbles.

Step 3—Casting and curing (Figure 2e). A release agent (Shenzhen Hongtu Silicone Technology Co., Ltd., Shenzhen, China) is applied to the inner surface before the degassed silicone mixture (Ecoflex 00-30, mixed at a 1:1 weight ratio) is slowly poured into the cavity and cured at 60 °C for 40 min.

Step 4—Demolding and sealing (Figure 2f). The silicone rubber is carefully peeled away from the mold, then the inner core is removed. After curing, the silicone body is carefully demolded, the inner core is removed, and a slit is cut through the end cover to insert the pneumatic supply tube (Smooth-On, Inc., Macungie, PA, USA). The tube joint is sealed with silicone-based sealant (Weiligoo V-1510, Dongguan Weiligu New Material Technology Co., Ltd., Dongguan, China), and a final layer of liquid silicone is applied to the end cap to ensure an airtight seal. To finish the end cap, liquid silicone is applied and left to cure.

How fiber threading and actuation work: A fiber thread is wound around the actuator body at regular intervals. When the air cavity is inflated, the inner cavity expands, leading to deformation of the actuator. The wound fibers constrain the radial expansion, which strengthens the axial elongation and overall structural integrity. Additionally, the geometric asymmetry of the air cavity design can lead to anisotropic deformation. The interplay of isotropic cavity expansion and anisotropic fiber constraint promotes a controlled bending motion along a predefined direction.

## 3. Static Model of Soft Actuator

Soft actuators are continuum structures with distributed degrees of freedom and geometrically nonlinear deformation; therefore, conventional rigid-link models are generally inadequate. The study of the constitutive model of a soft material plays a guiding role in establishing the fluid–structure coupling relationship of a pneumatic soft actuator. Owing to its hyperelastic characteristics, silicone rubber can undergo large-strain deformation—extending to multiples of its original dimensions—under mechanical stress. All the work done by the soft material under stress is converted into strain energy [21]. Upon removal of the applied stress, the material fully recovers to its original configuration. The soft actuator material is assumed to exhibit isotropic mechanical properties in the undeformed state, with the orientation of its long molecular chains being randomly and uniformly distributed [22]. A constitutive model was established based on the strain energy formulation, through which the constitutive relationship—linking stress to strain—was derived.

### 3.1. Constitutive Model of Soft Actuator

The soft actuator structure was composed of silicone rubber and winding fibers. Accordingly, both the silicone rubber matrix and the wound fibers were incorporated into the constitutive modeling framework of the soft actuator. The deformed part of the soft actuator was an isotropic silicone rubber material, and the cladding layer was an anisotropic winding fiber.

The constitutive behavior of silicone rubber was characterized using the Neo-Hookean model, a framework rooted in microscopic statistical thermodynamics [23,24]. This thermodynamic approach is underpinned by the molecular network structure, which enables hyperelastic behavior and large deformations [25]. As a hyperelastic material exhibiting isotropic properties and near-incompressible volumetric response during deformation, silicone rubber satisfies the fundamental assumptions of the Neo-Hookean constitutive formulation. The winding fiber of a soft actuator is an anisotropic material. Different strain energy expressions were used for the silicone structure and winding fiber of the soft actuator, where *W*(*gel*) and *W*(*fab*) correspond to the strain energies of the isotropic silicone rubber and the anisotropic wound fiber, respectively.

For the isotropic material, the Neo-Hookean strain energy density takes the form.

(1) $$W\left(gel\right)=\frac{\mu }{2}\left(tr\left(F^{T}F\right)-d\right)-\mu \log\left(J\right)+\frac{K}{2}\log^{2}\left(J\right)$$

(2) $$\mu =\frac{E}{2\left(1+\nu \right)}$$

(3) $$K=\frac{E\nu }{\left(1+\nu \right)\left(1-2\nu \right)}$$

The parameter *J* represents the elastic volume ratio (*J* = 1) under the incompressibility assumption adopted herein, and *d* is the model dimensionality—set to *d* = 3 for the present three-dimensional analysis [26]. The principal stretches are denoted by *K*, while *E* and *μ* correspond to Young’s modulus and Poisson’s ratio, respectively [27]. Within the Neo-Hookean framework, the strain energy density is formulated as a function of the invariant *tr*(*F^T^F*). Under the condition of volume conservation (*J* = 1), this expression reduces to.

(4) $$W\left(gel\right)=\frac{\mu }{2}\left(tr\left(F^{T}F\right)-3\right)$$

For an anisotropic material, the elastic strain energy *W*(*fab*) is determined by the deformation of the winding fiber. It should be noted that the incompressibility condition *J* = 1 is adopted as an idealization in the analytical derivation to keep the closed-form strain-energy expression tractable. In the finite element implementation, however, strict incompressibility cannot be enforced directly; instead, a nearly incompressible Neo-Hookean formulation is used, in which a finite bulk modulus *K* is retained and the volumetric strain-energy term (1/D_1_)(*J* − 1)^2^ penalizes deviations of the elastic volume ratio from unity. To experimentally validate the Neo-Hookean model parameters for the silicone rubber matrix used in this study, uniaxial tensile tests were conducted on Ecoflex 00-30 specimens prepared under identical processing conditions (1:1 base-to-curing-agent weight ratio, cured at 60 °C for 40 min). Standard dumbbell-shaped specimens (ASTM D412 Type C) were tested on a universal testing machine at a crosshead speed of 50 mm/min, and the nominal stress–strain response was recorded up to a stretch ratio of *λ* = 1.6. The experimental data were fitted to the Neo-Hookean model (Equation (4)) using a least-squares optimization routine. The fitted material parameters are: shear modulus *μ* = 0.068 MPa and bulk modulus *K* = 0.34 Mpa. The volumetric response is weakly compressible and closely approximates the incompressible behavior assumed in the analytical model, while avoiding the numerical difficulties associated with a fully incompressible formulation. The coefficient of determination R^2^ = 0.982 confirms that the Neo-Hookean model accurately captures the stress–strain behavior of Ecoflex 00-30 within the deformation range relevant to the present soft actuator. A detailed description of the parameter identification procedure and a comparison of the fitted curve with the experimental data can be found in our prior work [28]. In that previous study, the Neo-Hookean model was systematically compared with higher-order hyperelastic formulations, including the Yeoh, Ogden, and Mooney-Rivlin models, on the basis of uniaxial tensile test data of Ecoflex 00-30; the Neo-Hookean model was found to provide a comparable goodness of fit (R^2^ = 0.982) while requiring fewer material parameters. It should be noted that this model comparison was performed in the context of material characterization [28] and did not involve finite element simulations of the actuator investigated in the present study. A similar conclusion—that the Neo-Hookean model strikes an effective balance between accuracy and computational efficiency for soft pneumatic actuator simulations—has also been reported by Gariya et al. [29], who compared multiple hyperelastic models for soft actuator bending analysis. The Johnson–Cook model, a rate- and temperature-dependent plasticity formulation developed for the high-strain-rate inelastic response of metals, is not applicable to the quasi-static, recoverable, hyperelastic deformation of silicone rubber considered here and is therefore excluded from the constitutive comparison. The winding fiber can be modeled as a rod with an axial load. The cross-sectional area of the rod is *c*, the angle of the winding fiber in the axial direction is *α*_1_, and the initial direction *s*_1_ of the winding fiber can be expressed as:

(5) $$s_{1}=\left(0,\cos\alpha_{1},\sin\alpha_{1}\right)$$

$\bar{F}$ is the force in the axial load direction, and the winding fiber direction *S*_1_ under the force is expressed as.

(6) $$S_{1}=\bar{F}s_{1}$$

For a section of winding fiber with length *dl*_1_, the length change is *dx*_1_. The fiber stretches linearly under the force $\bar{F}$, and the strain energy *dW*(*fab*) of the fiber length *dl*_1_ is the area formed by the force–displacement curve. It can be expressed as:

(7) $$dW\left(fab\right)=\frac{1}{2}\bar{F}dx_{1}$$

(8) $$\bar{F}=E_{f}cQ_{1}$$

(9) $$Q_{1}=\frac{dx_{1}}{dl_{1}}$$

where *E_f_* is Young’s modulus of the fiber, *Q*_1_ is the axial strain of the fiber length *dl*_1_, and *dW*(*fab*) is expressed as:

(10) $$dW\left(fab\right)=\frac{1}{2}EcQ_{1}dx_{1}=\frac{1}{2}Ecdl_{1}Q_{1}^{2}$$

The strain energy *W*(*fab*) and strain energy density *W*(*dfab*) of the anisotropic material (winding fiber) are expressed as follows:

(11) $$W\left(fab\right)=\int_{0}^{L}\frac{1}{2}Ecdl_{1}Q_{1}^{2}=\frac{1}{2}EcLQ_{1}^{2}$$

(12) $$W\left(dfab\right)=\frac{1}{2}EQ_{1}^{2}$$

This is the strain energy under the condition that the soft actuator is wound with a single group of fibers. If the actuator is wound with several groups of fibers, the winding angles of the different groups are different. The corresponding strain-energy expression must be modified.

If the actuator is wound with two groups of fibers, the angle of the other group of fibers in the axial direction is *α*_2_; the initial direction *s*_2_ is expressed as:

(13) $$s_{2}=\left(0,\cos\alpha_{2},\sin\alpha_{2}\right)$$

(14) $$S_{2}=\bar{F}s_{2}$$

(15) $$dx_{2}=s_{2}dl_{2}-dl_{2}=\bar{F}S_{2}dl_{2}-dl_{2}$$

(16) $$Q_{2}=\frac{dx_{2}}{dl_{2}}=\bar{F}S_{2}-1$$

The strain energy density *W*(*dfab*) can be expressed as.

(17) $$W\left(dfab\right)=\frac{1}{2}EQ_{1}^{2}+\frac{1}{2}EQ_{2}^{2}$$

Similarly, when the actuator is wound with three groups of fibers, the angle of the third group of fibers in the axial direction is *α*_3_. We define the strain energy density *W*(*dfab*) as.

(18) $$W\left(dfab\right)=\frac{1}{2}EQ_{1}^{2}+\frac{1}{2}EQ_{2}^{2}+\frac{1}{2}EQ_{3}^{2}$$

The total strain energy of the soft actuator, *W*(*act*), is the sum of the contributions from the isotropic silicone rubber matrix and the anisotropic fiber reinforcement. *W*(*act*) can be expressed as follows:

(19) $$W\left(act\right)=a_{1}W\left(gel\right)+a_{2}W\left(dfab\right)$$

where *a*_1_ and *a*_2_ are the corresponding volume fractions.

### 3.2. Extension, Expansion and Twist Models of Soft Actuator

The isotropic material of the soft actuator has uniform stiffness. The actuator maintains a cuboid shape during inflation deformation. The isotropic material has an initial internal cavity length *L_n_*, initial external length *L_z_*, initial internal cavity width *K_n_* and initial external width *K_z_*. The isotropic material becomes the internal cavity length *l_n_* and external length *l_z_* during inflation deformation, the internal cavity width *k_n_* and the external width *k_z_*. The external anisotropic winding fiber has the initial length, *L_w_*, and the initial width, *K_w_*. We modeled and analyzed the extension, expansion, and twisting of the soft actuator.

**Figure 3:**
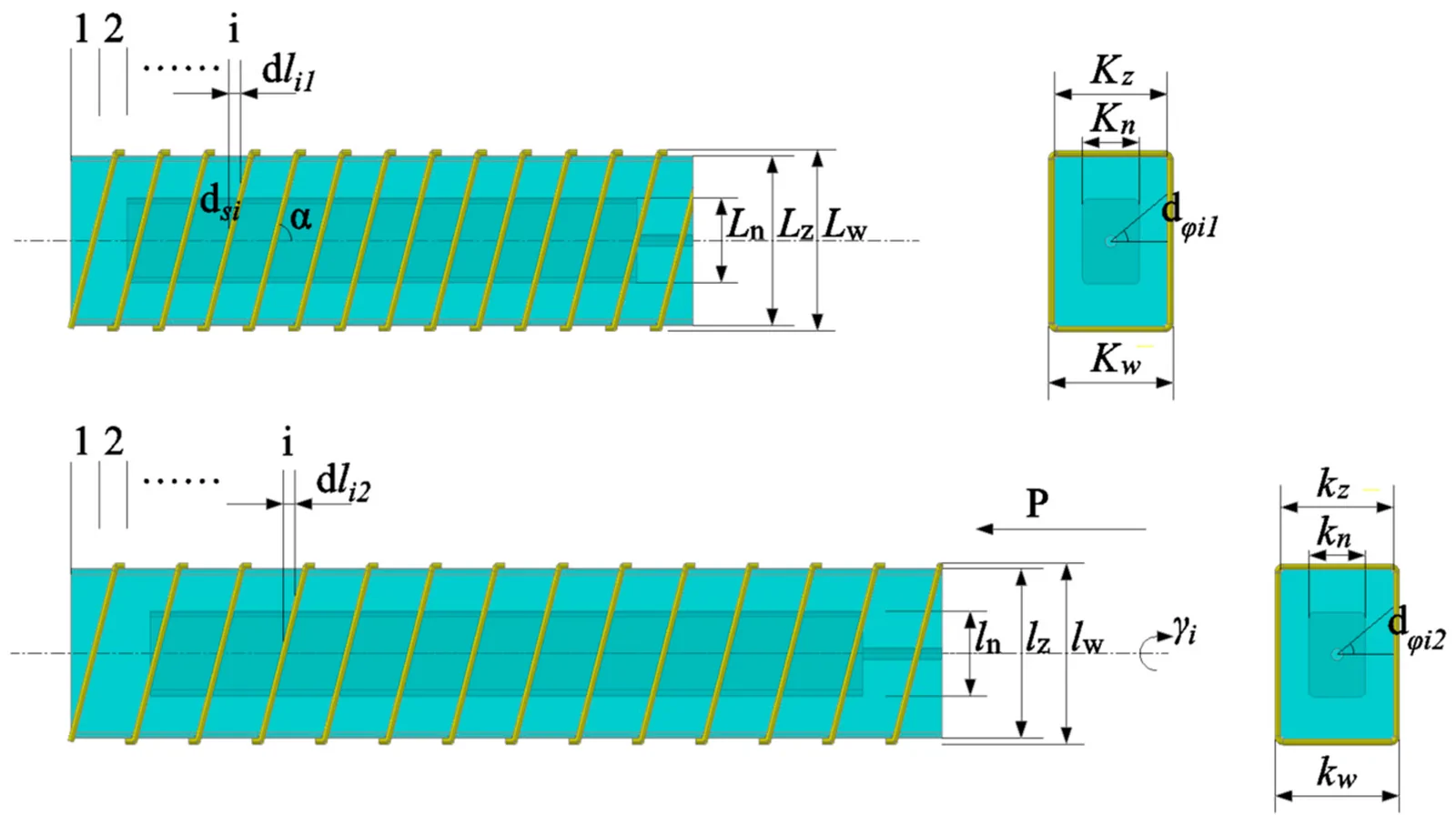
Soft actuator constrained by a single fiber.

The soft actuator’s deformation was limited by a single fiber, as presented in Figure 3. The solid and dotted lines denote the initial and deformed states of the soft actuator, respectively. We divided the soft actuator into several segments and used the *i*–th segment for the analysis.

As shown in Figure 3, *α* is the angle between the winding fiber and the axial direction of the soft actuator. The unit length of the winding fiber was *ds_i_*. Owing to the characteristics of the fiber, the length remained unchanged during the deformation of the soft actuator. *Dl_i_*_1_ and *dl_i_*_2_ are the lengths of *ds_i_* in the corresponding axial direction before and after deformation of the soft actuator, respectively. Before the deformation, the length of the *ds_i_* section of the soft actuator was *L_w_*_1_, the width was *K_W_*_1_, and the corresponding center angle was *dφ_i_*_1_. After deformation, the length of the *ds_i_* section of the soft actuator was *l_w_*_2_, the width was *k_W_*_2_, and the corresponding center angle was *dφ_i_*_2_. *ds_i_* and angle *α* can be expressed as:

(20) $$ds_{i}=\sqrt{dl_{i1}^{2}+{\left(\frac{L_{w1}+K_{w1}}{2}\right)}^{2}d\varphi_{i1}^{2}}=\sqrt{dl_{i2}^{2}+{\left(\frac{l_{w2}+k_{w2}}{2}\right)}^{2}d\varphi_{i2}^{2}}$$

(21) $$\tan\alpha =\frac{\frac{L_{w1}+K_{w1}}{2}}{dl_{i1}}$$

(22) $${\left(\frac{dl_{i2}}{dl_{i1}}\right)}^{2}\cos^{2}\alpha +{\left(\frac{\frac{l_{w2}+k_{w2}}{2}d\varphi_{i2}}{\frac{L_{w1}+K_{w1}}{2}d\varphi_{i1}}\right)}^{2}\sin^{2}\alpha =1$$

During the deformation of the *i*th segment of the soft actuator, the fiber length *ds* can be expressed by the angle *dφ_i_*. The number of fiber winding turns, *n_i_*, during actuator deformation can be expressed as:

(23) $$n_{i}=\frac{\varphi_{i}}{2\pi }$$

The isotropic material of the soft actuator was uniformly deformed. After deformation, the rotation angle of a single fiber length *ds_i_* along the central axis is *γ_i_*.

(24) $$\frac{d\varphi_{i2}}{d\varphi_{i1}}=\frac{d\varphi_{i1}+\gamma_{i}}{d\varphi_{i1}}$$

(25) $${\left(\frac{dl_{i2}}{dl_{i1}}\right)}^{2}\cos^{2}\alpha +{\left(\frac{\frac{l_{w2}+k_{w2}}{2}\left(d\varphi_{i1}+\gamma_{i}\right)}{\frac{L_{w1}+K_{w1}}{2}d\varphi_{i1}}\right)}^{2}\sin^{2}\alpha =1$$

The total rotation angle of a single fiber around the axis was γ:

(26) $$\gamma =\sum_{i=1}^{n}\gamma_{i}$$

**Figure 4:**
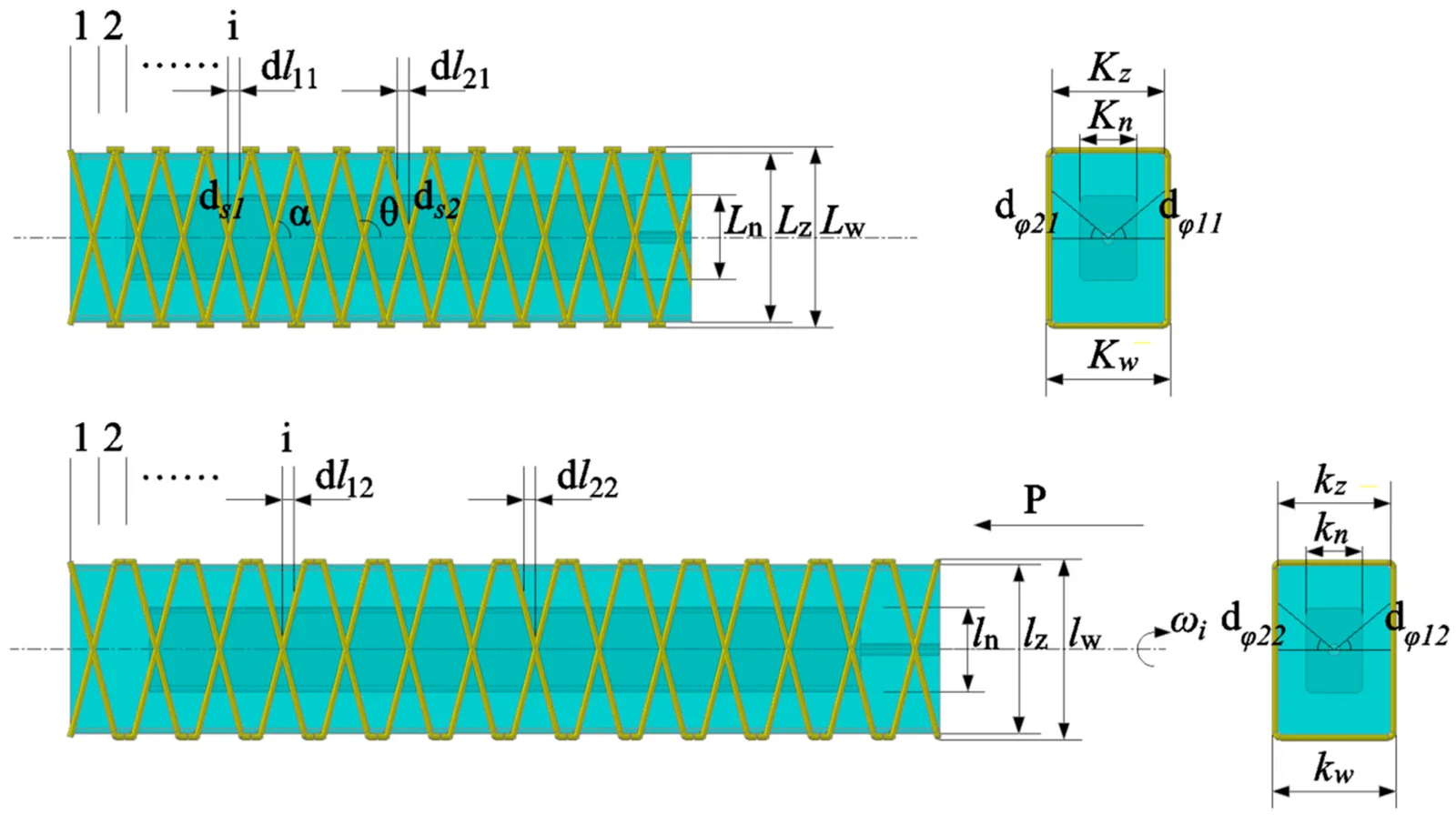
Soft actuator constrained by two symmetrical winding fibers.

We analyzed the radial constraint model of a single fiber. Using the same analysis method, two fibers were wound symmetrically around the actuator. We divided the soft actuator into several segments and used the *i*th segment for the analysis. As shown in Figure 4, the angles between the two fibers and the central axis of the soft actuator were *α* and *θ*, respectively. The fibers were wound symmetrically around the actuator.

(27) α+θ=π

The unit length of the winding fiber was *ds_i_*. The length of the first fiber was *ds*_1_ and the length of the second fiber was *ds*_2_, such as:

(28) $$ds_{1}=ds_{2}$$

Owing to the characteristics of the fiber, the length remained unchanged as the soft actuator undergoes deformation. *Dl*_11_ and *dl*_12_ are the lengths of *ds*_1_ in the corresponding axial direction before and after deformation of the soft actuator. *Dl*_21_ and *dl*_22_ are the lengths of *ds*_1_ in the corresponding axial direction before and after deformation of the soft actuator. Before deformation, the lengths of the *ds*_1_ and *ds*_2_ sections of the soft actuator were *L_w_*_1_, the width was *K_W_*_1_, and the corresponding center angles were *dφ*_11_ and *dφ*_21_. After deformation, the lengths of the *ds*_1_ and *ds*_2_ sections of the soft actuator were *l_w_*_2_, the width was *k_W_*_2_, and the corresponding center angles were *dφ*_12_ and *dφ*_22_. The rotation angle of the first fiber around the axis is γ_1_ and that of the second fiber around the axis is γ_2_. The rotation angle of the two fibers around the axis is given by:

(29) $$\omega_{i}=\gamma_{1}-\gamma_{2}$$

The *ds*_1_ and *ds*_2_ can be expressed as:

(30) $$ds_{1}=\sqrt{dl_{11}^{2}+{\left(\frac{L_{w1}+K_{w1}}{2}\right)}^{2}d\varphi_{11}^{2}}=\sqrt{dl_{12}^{2}+{\left(\frac{l_{w2}+k_{w2}}{2}\right)}^{2}d\varphi_{12}^{2}}$$

(31) $$ds_{2}=\sqrt{dl_{21}^{2}+{\left(\frac{L_{w1}+K_{w1}}{2}\right)}^{2}d\varphi_{21}^{2}}=\sqrt{dl_{22}^{2}+{\left(\frac{l_{w2}+k_{w2}}{2}\right)}^{2}d\varphi_{22}^{2}}$$

(32) $$\tan\alpha =\frac{\frac{L_{w1}+K_{w1}}{2}}{dl_{11}}$$

(33) $${\left(\frac{dl_{12}}{dl_{11}}\right)}^{2}\cos^{2}\alpha +{\left(\frac{\frac{l_{w2}+k_{w2}}{2}d\varphi_{12}}{\frac{L_{w1}+K_{w1}}{2}d\varphi_{11}}\right)}^{2}\sin^{2}\alpha =1$$

(34) $${\left(\frac{dl_{22}}{dl_{21}}\right)}^{2}\cos^{2}\theta +{\left(\frac{\frac{l_{w2}+k_{w2}}{2}d\varphi_{22}}{\frac{L_{w1}+K_{w1}}{2}d\varphi_{21}}\right)}^{2}\sin^{2}\theta =1$$

Similarly, the isotropic material of the soft actuator was uniformly deformed. The deformations of the two fibers per unit length were the same.

(35) $$\frac{d\varphi_{12}}{d\varphi_{11}}=\frac{d\varphi_{11}+\gamma_{1}}{d\varphi_{11}}$$

(36) $$\frac{d\varphi_{22}}{d\varphi_{21}}=\frac{d\varphi_{21}+\gamma_{2}}{d\varphi_{21}}$$

(37) $${\left(\frac{dl_{12}}{dl_{11}}\right)}^{2}\cos^{2}\alpha +{\left(\frac{\frac{l_{w2}+k_{w2}}{2}\left(d\varphi_{11}+\gamma_{1}\right)}{\frac{L_{w1}+K_{w1}}{2}d\varphi_{11}}\right)}^{2}\sin^{2}\alpha =1$$

(38) $${\left(\frac{dl_{22}}{dl_{21}}\right)}^{2}\cos^{2}\theta +{\left(\frac{\frac{l_{w2}+k_{w2}}{2}\left(d\varphi_{21}+\gamma_{2}\right)}{\frac{L_{w1}+K_{w1}}{2}d\varphi_{21}}\right)}^{2}\sin^{2}\theta =1$$

(39) $$\gamma_{1}=d\varphi_{11}\left[\frac{\left(L_{w1}+K_{w1}\right)\sqrt{1-{\left(\frac{dl_{12}}{dl_{11}}\right)}^{2}\cos^{2}\alpha }}{\left(l_{w2}+k_{w2}\right)\sin\alpha }-1\right]$$

(40) $$\gamma_{2}=d\varphi_{21}\left[\frac{\left(L_{w1}+K_{w1}\right)\sqrt{1-{\left(\frac{dl_{22}}{dl_{21}}\right)}^{2}\cos^{2}\theta }}{\left(l_{w2}+k_{w2}\right)\sin\theta }-1\right]$$

(41) α+θ=π

(42) $$\omega_{i}=\gamma_{1}-\gamma_{2}=0$$

(43) $$\omega =\sum_{i=1}^{n}\omega_{i}=0$$

In summary, when two or more fibers are symmetrically wound around the soft actuator, the soft actuator is not twisted in the axial direction.

We continued to analyze the extension and expansion of soft actuators. The soft actuator was made of an isotropic material. Axial, circumferential, and radial stretching occurred during the deformation of the soft actuator. The three tensile coefficients were defined. The axial stretching coefficient per unit length was *λ*_1_, the circumferential stretching coefficient was *λ*_2_, and the radial stretching coefficient was *λ*_3_. The tensor invariants *tr*(*F^T^F*) of the soft actuator are expressed as:

(44) $$tr\left(F^{T}F\right)=\lambda_{1}^{2}+\lambda_{2}^{2}+\lambda_{3}^{2}$$

The strain energy of the Neo–Hookean model of isotropic materials can be expressed as.

(45) $$W\left(gel\right)=\frac{\mu }{2}\left(\lambda_{1}^{2}+\lambda_{2}^{2}+\lambda_{3}^{2}-3\right)$$

The isotropic material of the soft actuator has uniform stiffness. The actuator maintained a cuboid shape during inflation deformation. The radial stretching coefficient *λ*_3_ is expressed as follows:

(46) $$\lambda_{3}=\frac{l_{z}-l_{n}}{L_{z}-L_{n}}=\frac{k_{z}-k_{n}}{K_{z}-K_{n}}$$

The soft actuator was wound by the fiber confinement layer, and the circumferential stretching coefficient was negligible.

(47) $$\lambda_{2}=1$$

The isotropic material of the soft actuator was incompressible. *λ*_1,_ *λ*_2_, and *λ*_3_ are expressed as follows:

(48) $$\lambda_{1}^{2}\cdot \lambda_{2}^{2}\cdot \lambda_{3}^{2}=1$$

The axial stretching coefficient per unit length of the soft actuator is *λ*.

(49) $$\lambda_{1}=\lambda ,\;\lambda_{2}=1,\;\lambda_{3}=\frac{1}{\lambda }=\frac{l_{z}-l_{n}}{L_{z}-L_{n}}$$

The deformation gradient matrix F of the soft actuator can be expressed as:

(50) $$F=\left\{\begin{array}{l} \lambda \;0\;0\\ 0\;1\;0\\ 0\;0\;\frac{1}{\lambda } \end{array}\right\}$$

The Cauchy stress, *σ*(*gel*), of an isotropic material can be expressed using the strain energy expression.

(51) $$\begin{array}{ll} \sigma \left(gel\right) & =\frac{\partial W\left(gel\right)}{\partial \lambda }-pF^{T}=\frac{\partial \left(\frac{\mu }{2}\left(\lambda_{1}^{2}+\lambda_{2}^{2}+\lambda_{3}^{2}-3\right)\right)}{\partial \lambda }-pF^{T}\\ & =\frac{\mu }{2}\frac{\partial }{\partial \lambda }\left(\lambda^{2}+\frac{1}{\lambda^{2}}-2\right)-pF^{T}=\mu \left(\lambda -\frac{1}{\lambda^{3}}\right)-pF^{T} \end{array}$$

The deformation of the anisotropic material of the soft actuator in the circumferential and radial directions is negligible. The Cauchy stress *σ*(*fab*) of an anisotropic material is the unit strain energy of the fibers in the axial direction, such as:

(52) $$\sigma \left(fab\right)=\frac{\partial W\left(fab\right)}{\partial cL}s_{n}⊗s_{n}=W\left(mfab\right)s_{n}⊗s_{n}$$

where *s_n_* is the axial direction of each fiber. The total Cauchy stress *σ* of the soft actuator is expressed as:

(53) $$\sigma =\sigma \left(gel\right)+\sigma \left(fab\right)=\mu \left(\lambda -\frac{1}{\lambda^{3}}\right)-pF^{T}+W\left(mfab\right)s_{n}⊗s_{n}$$

According to the structure of the soft actuator and the Cauchy equilibrium equation, it can be expressed as:

(54) $$P=\frac{1}{2}\int_{l_{n}}^{l_{z}}\frac{\sigma {\left(gel\right)}_{\lambda 3}}{l}dl+\frac{1}{2}\int_{l_{z}}^{l_{w}}\frac{\sigma {\left(fab\right)}_{\lambda 3}}{l}dl$$

(55) $$\frac{d\sigma_{\lambda 3}}{dl}=\frac{1}{2}\frac{\sigma_{\lambda 3}}{l}$$

where *P* is the pressure in the air cavity of the isotropic material, *σ_λ_*_3_ is the radial Cauchy stress of the soft actuator, *σ*(*gel*)*_λ_*_3_ is the radial Cauchy stress of the isotropic material, and *σ*(*fab*)*_λ_*_3_ is the radial Cauchy stress of the anisotropic material. When the soft actuator only extended and expanded, there was no external axial force or torque applied to it. The axial Cauchy stress of the soft actuator is *σ_λ_*_1_. Axial load N is expressed as follows:

(56) $$N=\frac{1}{2}\int_{l_{n}}^{l_{z}}\sigma {\left(gel\right)}_{\lambda 1}dl+\frac{1}{2}\int_{l_{z}}^{l_{w}}\sigma {\left(fab\right)}_{\lambda 1}dl=Pl_{w}k_{w}$$

### 3.3. Bending Model of Soft Actuator

When the flexible actuator is bent, the circumferential strain is negligible owing to the limitation of the external winding fibers. The deformation of the soft actuator was mainly affected by axial strain. The radial Cauchy stress, *σ_λ_*_3_, was much smaller than the axial Cauchy stress, *σ_λ_*_1_. The deformation of the soft actuator was mainly affected by the axial stress. The radial stress and deformation of the soft actuator were ignored.

The bending of the soft actuator was performed using multiple moments: the moment generated by the compressed air pressure around the endpoint in the air cavity of the soft actuator, and moments generated by isotropic and anisotropic materials of soft actuators.

**Figure 5:**
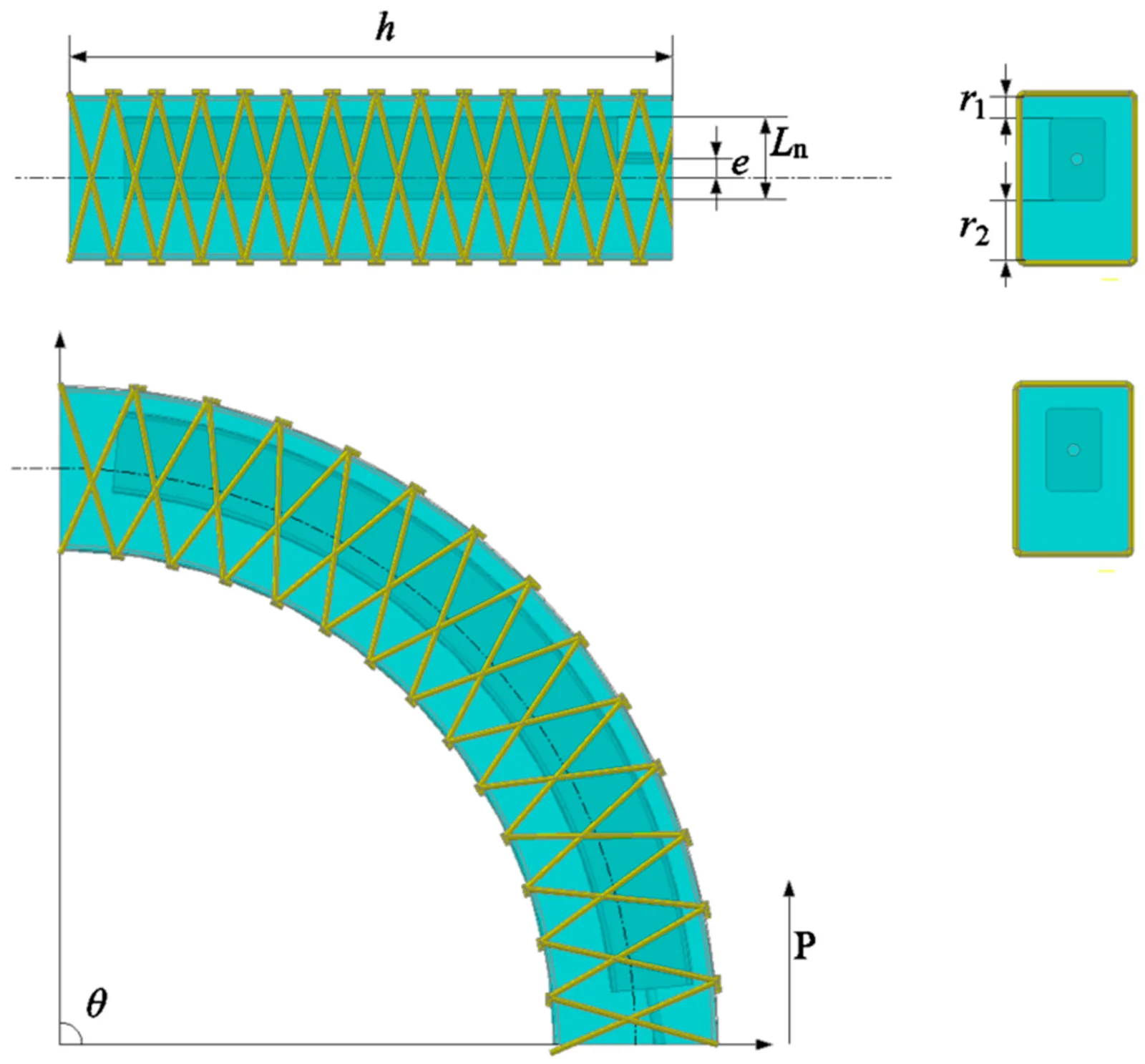
Bending deformation of soft actuator.

As shown in Figure 5, four moments acted on the soft actuator. The moment *M_p_* of the compressed air pressure *P* acts on the air cavity of the actuator, moment *M_ns_* of the upper half of the isotropic material, moment *M_nx_* of the lower half of the isotropic material, and moment *M_w_* of the anisotropic material. The moment of the soft actuator can be expressed as:

(57) $$M_{p}=M_{ns}+M_{nx}+M_{w}$$

We analyzed the air pressure moment *M_p_*. The axial force *F_p_* is expressed as follows:

(58) $$F_{p}=\left(P-P_{atm}\right)S=\left(P-P_{atm}\right)k_{n}\bar{l_{n}}\sin\theta$$

(59) $$d\bar{l_{n}}=l_{n}\sin\theta d\theta$$

(60) $$dF_{p}=\left(P-P_{atm}\right)dS=\left(P-P_{atm}\right)k_{n}d\bar{l_{n}}\sin\theta$$

(61) $$dF_{p}=\left(P-P_{atm}\right)dS=\left(P-P_{atm}\right)k_{n}l_{n}\sin^{2}\theta d\theta$$

where *h* is the length of the air cavity of the soft actuator, *θ* is the angle at which the actuator is bent, *S* is the cross-sectional area of the air cavity, *P_atm_* is the atmospheric pressure, and $\bar{l_{n}}$ is the component of *l_n_* during deformation.

The actuator is divided into several segments, and the air pressure moment *M_p_* can be expressed as:

(62) $$M_{p}=\left(P-P_{atm}\right)l_{n}k_{n}\int_{0}^{\frac{\pi }{2}}\lambda_{1}h\cos\theta \;\sin^{2}\theta d\theta$$

The moments of the isotropic and anisotropic materials of the soft actuator can be expressed using the Cauchy stress derived above.

(63) $$M_{ns}=\frac{1}{2}\int_{0}^{r_{1}}\left(\int_{0}^{\frac{\pi }{2}}\lambda_{1}h\sigma {\left(gel,1\right)}_{\lambda 1}\left(l_{n}\left(k_{z}-k_{n}\right)+k_{z}r\cos\theta \right)d\theta \right)dr$$

(64) $$M_{nx}=\frac{1}{2}\int_{0}^{r_{2}}\left(\int_{0}^{\frac{\pi }{2}}\lambda_{1}h\sigma {\left(gel,2\right)}_{\lambda 1}\left(l_{n}\left(k_{z}-k_{n}\right)+k_{z}r\cos\theta \right)d\theta \right)dr$$

(65) $$M_{w}=\int_{0}^{\frac{\pi }{2}}\lambda_{1}h\sigma {\left(fab\right)}_{\lambda 1}\left(l_{z}\left(k_{w}-k_{z}\right)+k_{w}\left(l_{w}-l_{z}\right)\right)d\theta$$

(66) $$r_{i}=l_{z}-l_{n}$$

where *r_i_* is the thickness of the isotropic material in the upper and lower halves, *σ*(*gel*,1)*_λ_*_1_ is the axial Cauchy stress in the upper half of the isotropic material, *σ*(*gel*,2)*_λ_*_1_ is the axial Cauchy stress in the lower half of the isotropic material, and *σ*(*fab*)*_λ_*_1_ is the axial Cauchy stress in the anisotropic material.

The following expression describes how the bending angle *θ* of the soft actuator varies with the input air pressure *P*:

(67) $$\begin{array}{ll} P= & \frac{1}{2}\left(\begin{array}{l} \frac{\int_{0}^{r_{1}}\left(\int_{0}^{\frac{\pi }{2}}\sigma {\left(gel,1\right)}_{\lambda 1}\left(l_{n}\left(k_{z}-k_{n}\right)+k_{z}r\cos\theta \right)d\theta \right)dr}{l_{n}k_{n}\int_{0}^{\frac{\pi }{2}}\cos\theta \;\sin^{2}\theta d\theta }\\ +\frac{\int_{0}^{r_{2}}\left(\int_{0}^{\frac{\pi }{2}}\sigma {\left(gel,2\right)}_{\lambda 1}\left(l_{n}\left(k_{z}-k_{n}\right)+k_{z}r\cos\theta \right)d\theta \right)dr}{l_{n}k_{n}\int_{0}^{\frac{\pi }{2}}\cos\theta \;\sin^{2}\theta d\theta } \end{array}\right)\\ & +\frac{\int_{0}^{\frac{\pi }{2}}\sigma {\left(fab\right)}_{\lambda 1}\left(l_{z}\left(k_{w}-k_{z}\right)+k_{w}\left(l_{w}-l_{z}\right)\right)d\theta }{l_{n}k_{n}\int_{0}^{\frac{\pi }{2}}\cos\theta \;\sin^{2}\theta d\theta }+P_{atm} \end{array}$$

## 4. Finite Element Simulation of Soft Actuator Behavior

### 4.1. Finite Element Modeling

The analytical framework developed in Section 3 provides the constitutive foundation for the finite element simulations. The workflow connecting the analytical model to the numerical implementation proceeds as follows:

Step 1—material constitutive definition. The Neo-Hookean strain energy density function (Equations (1)–(4)) is used to define the hyperelastic behavior of the isotropic silicone rubber matrix, with the experimentally fitted parameters (*μ* = 0.068 MPa, *K* = 0.34 MPa; see Section 3.1) assigned as material properties in COMSOL Multiphysics (version 5.6). Consistent with the analytical treatment in Section 3.1, the Neo-Hookean model is implemented in its compressible (nearly incompressible) form, in which the fitted finite bulk modulus *K* governs the volumetric response.

Step 2—fiber reinforcement definition. The anisotropic strain energy formulation for the wound fibers (Equations (7)–(12)) is implemented to capture the directional reinforcing effect. The fiber winding angle(s) and the corresponding strain energy density *W*(*fab*) (Equations (11) and (12) for single fiber, or Equations (17) and (18) for multiple fiber groups) are specified as an anisotropic material layer.

Step 3—geometry and mesh. The actuator geometry is constructed with the dimensions defined in Section 2. The model is discretized using quadratic tetrahedral elements with local refinement in the cavity region.

Step 4—load and boundary conditions. A fixed constraint is applied at one end. The internal pressure *P* is applied as a prescribed boundary load on the cavity wall surfaces.

Step 5—solution. The nonlinear equilibrium equations are solved using the Newton–Raphson method. The total Cauchy stress σ (Equation (53)), the deformation gradient F, and the bending angle *θ* (Equation (67)) are computed as output variables for each pressure increment.

The finite element method provides a numerical simulation framework capable of handling coupled multi-physics problems involving solid mechanics, fluid dynamics, and other physical domains. The soft actuator bends due to the combined effects of the isotropic silicone rubber matrix and the anisotropic constraints of the wound fibers [30]. Since direct measurement of internal stresses and strains in the silicone elastomer and reinforcing fibers is extremely difficult, numerical simulation is used as an alternative approach. Therefore, the stress distribution and deformation response of the soft actuator were analyzed using the finite element method.

**Figure 6:**
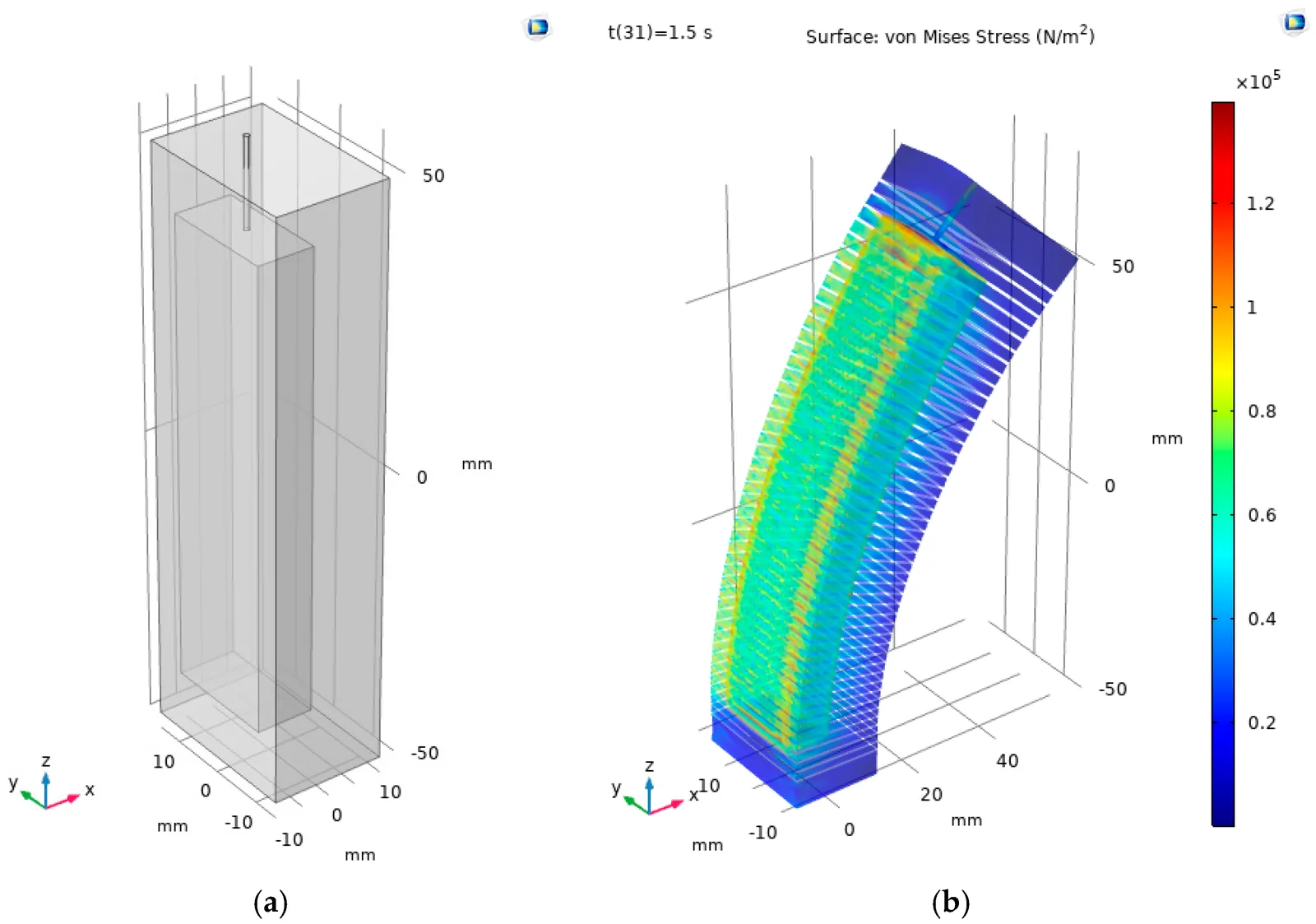
(**a**) Finite element mesh of the actuator; (**b**) bending stress distribution.

As illustrated in Figure 6a, the finite element simulations were performed using COMSOL Multiphysics. The solid mechanics module was used to model the hyperelastic deformation of the silicone rubber matrix and the anisotropic response of the wound fibers. The internal air pressure *P* was applied as a prescribed boundary load on the inner surfaces of the air cavity; no fluid domain was modeled and no fluid–structure interaction was involved, as the pressure is specified directly and neither airflow dynamics nor transient pressure evolution is investigated [31].

The actuator model was meshed using quadratic tetrahedral elements (Lagrange elements) in COMSOL Multiphysics. Local mesh refinement was applied to the air cavity region and the fiber-reinforced layer to resolve the expected stress gradients. The minimum element size in the cavity region was set to 0.3 mm and the maximum element size in the outer strain layer to 1.2 mm, resulting in approximately 35,000 tetrahedral elements and approximately 52,000 degrees of freedom for the full model. A mesh convergence study was performed by progressively halving the minimum element size (0.6 mm, 0.3 mm, 0.15 mm). The computed bending angle at *P* = 0.045 MPa changed by less than 1.5% between the 0.3 mm and 0.15 mm meshes, confirming that the 0.3 mm mesh provides mesh-independent results. The actuator was fixed at one end (zero displacement boundary condition: u = v = w = 0). No symmetry condition was applied, as the asymmetric cavity geometry requires a full three-dimensional model. The nonlinear equilibrium equations were solved using the Newton–Raphson method with a relative tolerance of 1 × 10^−3^ and an absolute tolerance of 1 × 10^−4^ (COMSOL default settings). The parametric pressure sweep was performed from *P* = 0 to 0.045 MPa in 0.005 MPa increments (10 load steps), with the solution at each step used as the initial guess for the next step to improve convergence. The present simulation involves no discretized fluid domain; therefore, the ALE formulation, moving-mesh treatment, and fluid compressibility modeling are not applicable.

The Neo-Hookean material parameters used in the finite element simulation were obtained from the experimental uniaxial tensile characterization of Ecoflex 00-30 described in Section 3.1, ensuring that the numerical model is grounded in directly measured material behavior [28]. While the anisotropic fiber reinforcement constrained radial expansion, directing deformation primarily along the axial direction, the cavity pressure and the eccentric geometry jointly drive the bending response. In the following, we examine how the stress and deformation change across three design variations.

(1) Using this model, we investigated three design parameters: winding fiber density, air cavity offset distance, and cavity cross-sectional geometry.
(2) How far away the air cavity is positioned from the surface impacts the level of stress and deformation.
(3) How the geometry of air cavities affects the stress and deformation behavior.

The study of how the soft actuator is deformed after inflation is detailed in Figure 6b. In the next section, we will look at how the stress state of the rubber is used as a way to characterize its deformation behavior. In particular, the stress and deformation patterns are analyzed on several surfaces that are aligned with the bending direction of the actuator. The surface closest to the air cavity is the most stressed layer. On the other hand, the surface furthest from the air cavity is the least stressed.

**Figure 7:**
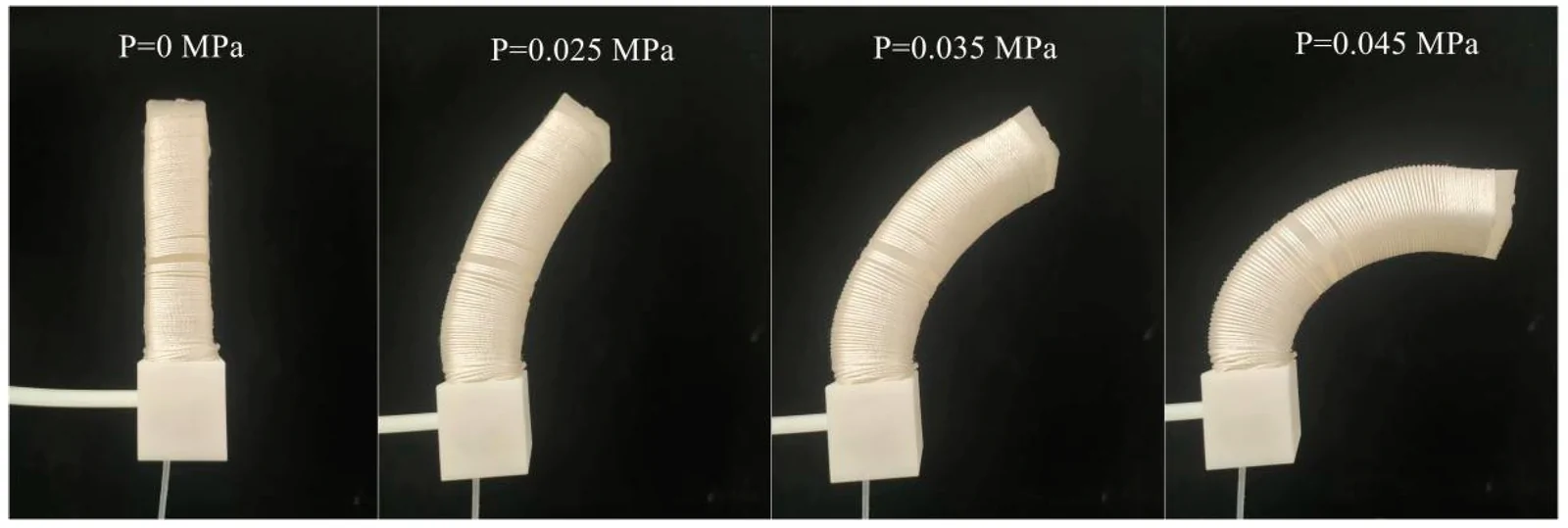
Fiber-reinforced soft actuator bending deformation process.

The simulated bending angles were validated experimentally. As Figure 7 shows, the bending angle increased monotonically with the input pressure *P*. When the input air pressure *P* = 0.045 MPa, the bending angle of the actuator is 90°. The bending deformation characteristics of the fiber-reinforced soft actuator are mainly affected by its structural parameters. By changing its structural parameters in the finite element simulation analysis, the distribution inside the actuator under different structural parameters can be analyzed. Based on the bending deformation principle of the soft actuator, in the finite element analysis, the outer fiber winding density, the distance of the air cavity from the central axis, and the shape of the air cavity are respectively changed to analyze and study the stress distribution and stress changes in its strain layer and restriction layer.

### 4.2. Influence of Winding Fiber Density on Actuator Deformation

**Figure 8:**
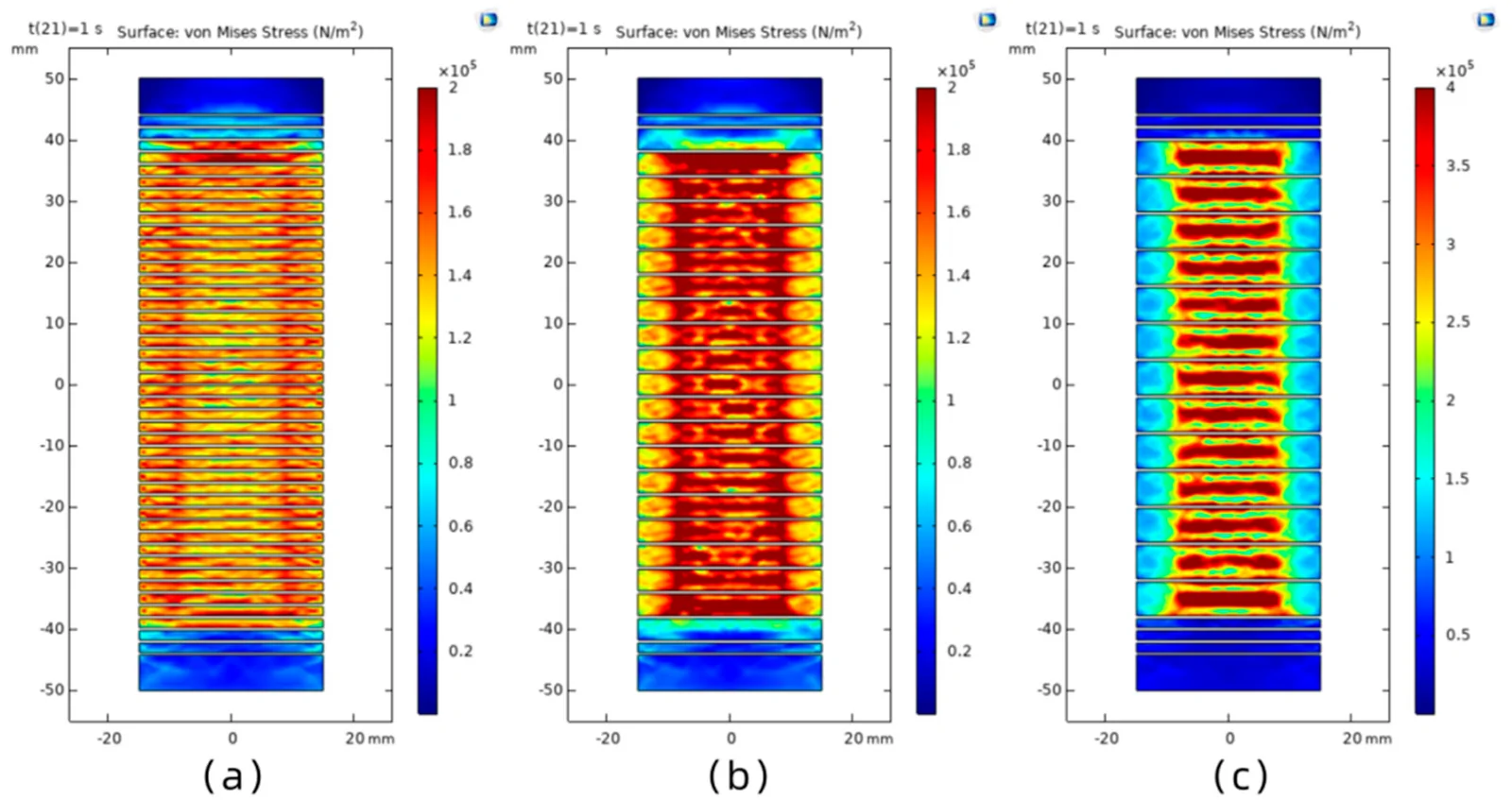
Strain-layer stress at three winding fiber densities: (**a**) high; (**b**) medium; and (**c**) low.

The stress orientation on the strain layer surface is strongly influenced by the density of windings of the fiber, as shown in Figure 8. In areas of high fiber density, the stress field across the strain layer is relatively uniform, with minor concentration observed along the contour of the air cavity. When the fiber density goes down, stress becomes more localized at both individual fibers and between them. This localized stress concentration creates a characteristic “bulging” pattern on the surface of the strain layer, which is accompanied by non-uniform deformation and a reduction in tensile strain in this layer.

**Figure 9:**
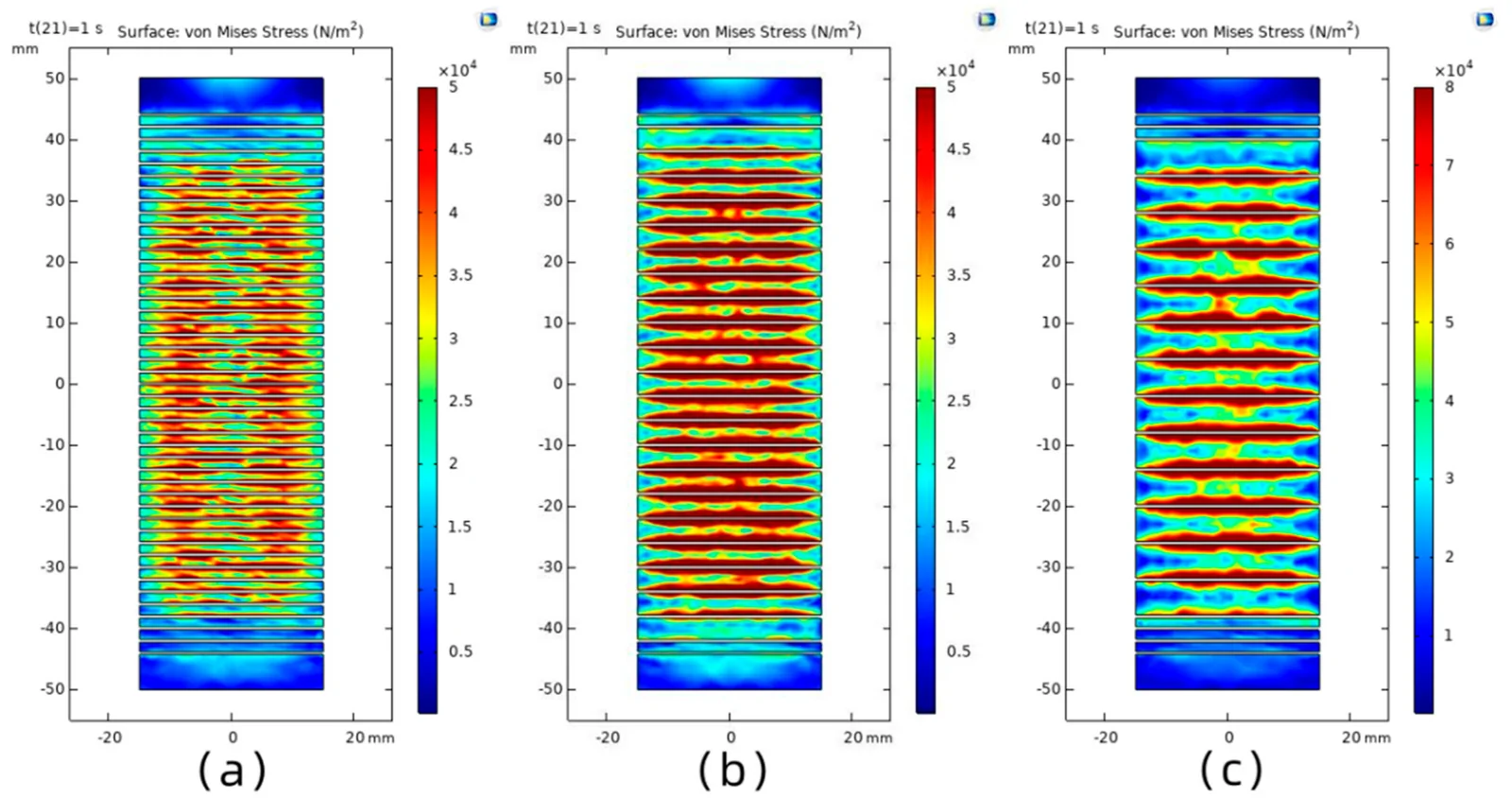
Limiting-layer stress under the same three winding fiber densities: (**a**) high; (**b**) medium; and (**c**) low.

The same dependence on fiber density is found for the limiting layer (Figure 9). At low fiber density, stress concentrates at the fiber locations, reducing the tensile deformation of the outermost layer.

**Figure 10:**
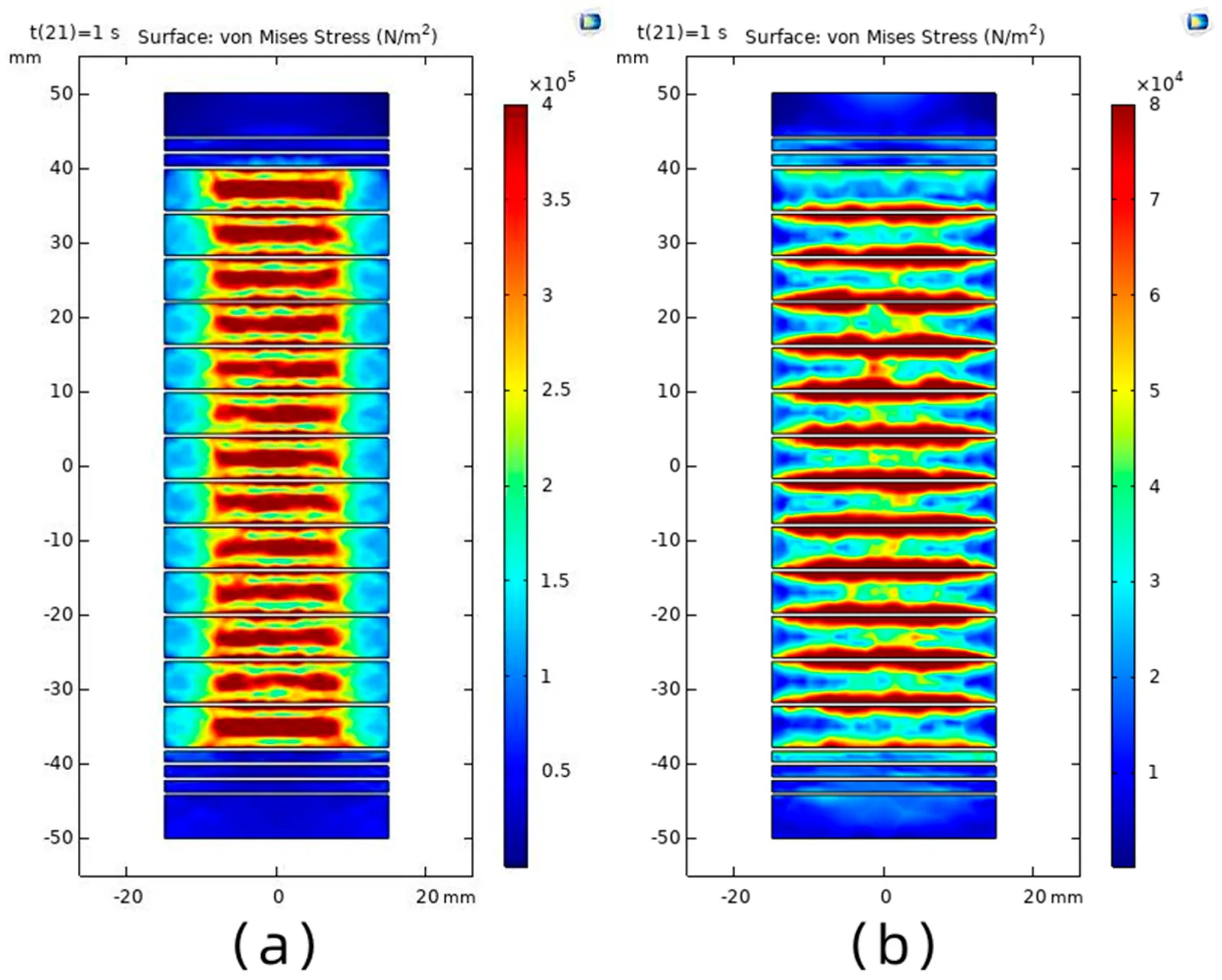
Comparison of strain-layer (**a**) and limiting-layer (**b**) stress patterns at low fiber density.

In conditions with low fiber density, both layers experience tensile deformation, but the areas of stress concentration differ significantly (Figure 10). In the strain layer, stress concentrates from axial expansion along the centerline and from tension at the fiber gaps in the silicone rubber. In the limiting layer, by contrast, the dominant source is radial expansion along the centerline, with the fiber windings introducing localized compressive stress.

The density of fiber winding is a key factor in shaping the bending behavior of soft actuators. At high fiber densities, the stress fields over both the strain and limiting layers become increasingly uniform. In contrast, the uneven distribution of stress, combined with significant local variations, weakens the stability of the strain layer deformation, which in turn affects the actuator’s bending behavior.

**Figure 11:**
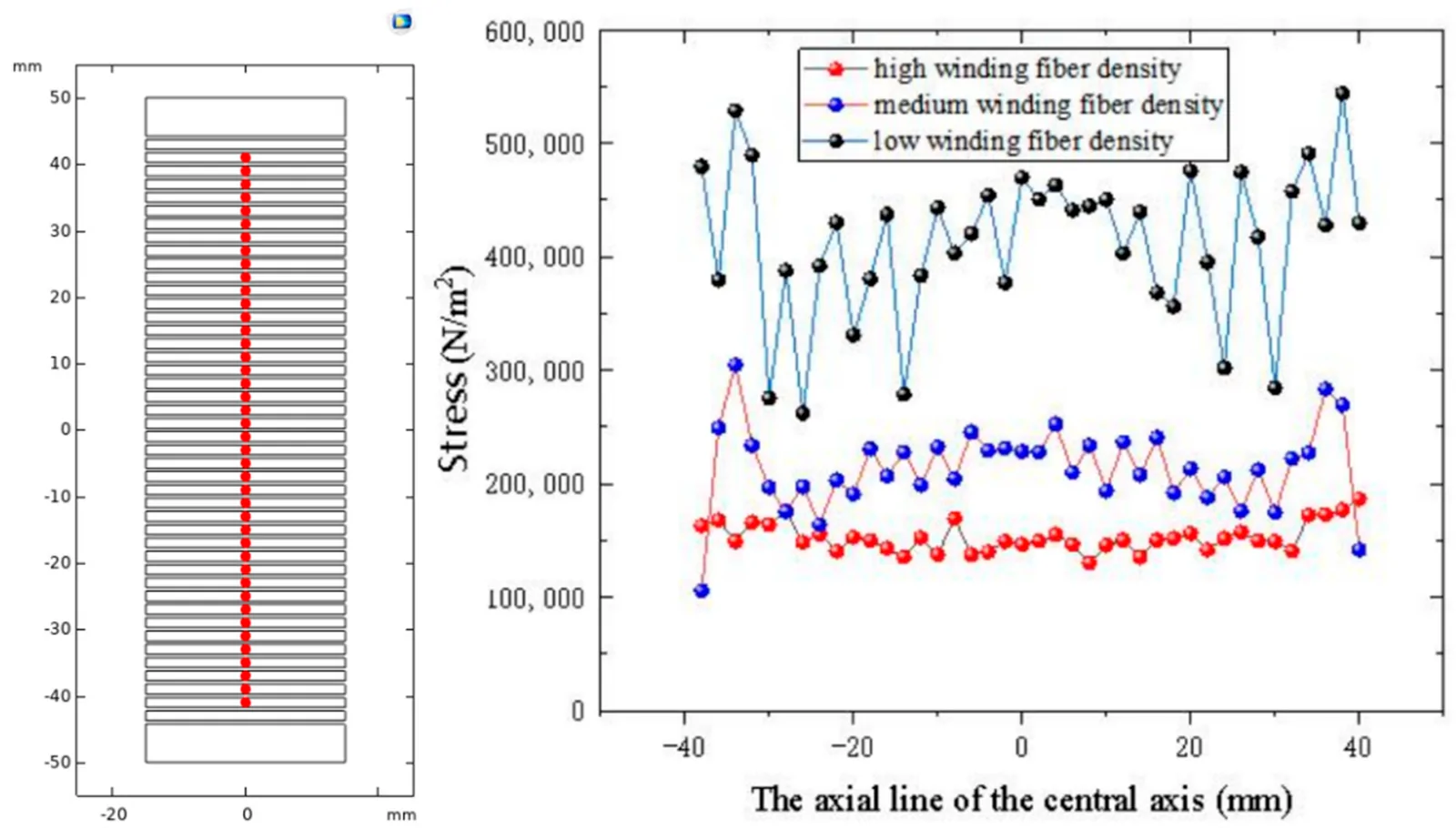
The stress curve on the axial line of the central axis of the strain layer with different winding fiber densities.

At high fiber density, the stress along the central axis of the strain layer remained relatively uniform (Figure 11). As fiber density decreased, the stress level rose and fluctuated more strongly: with wider spacing between adjacent fibers, the silicone rubber expanded freely under cavity pressure between the constrained points, creating large stress gradients at the fiber intervals and forming local bulges.

**Figure 12:**
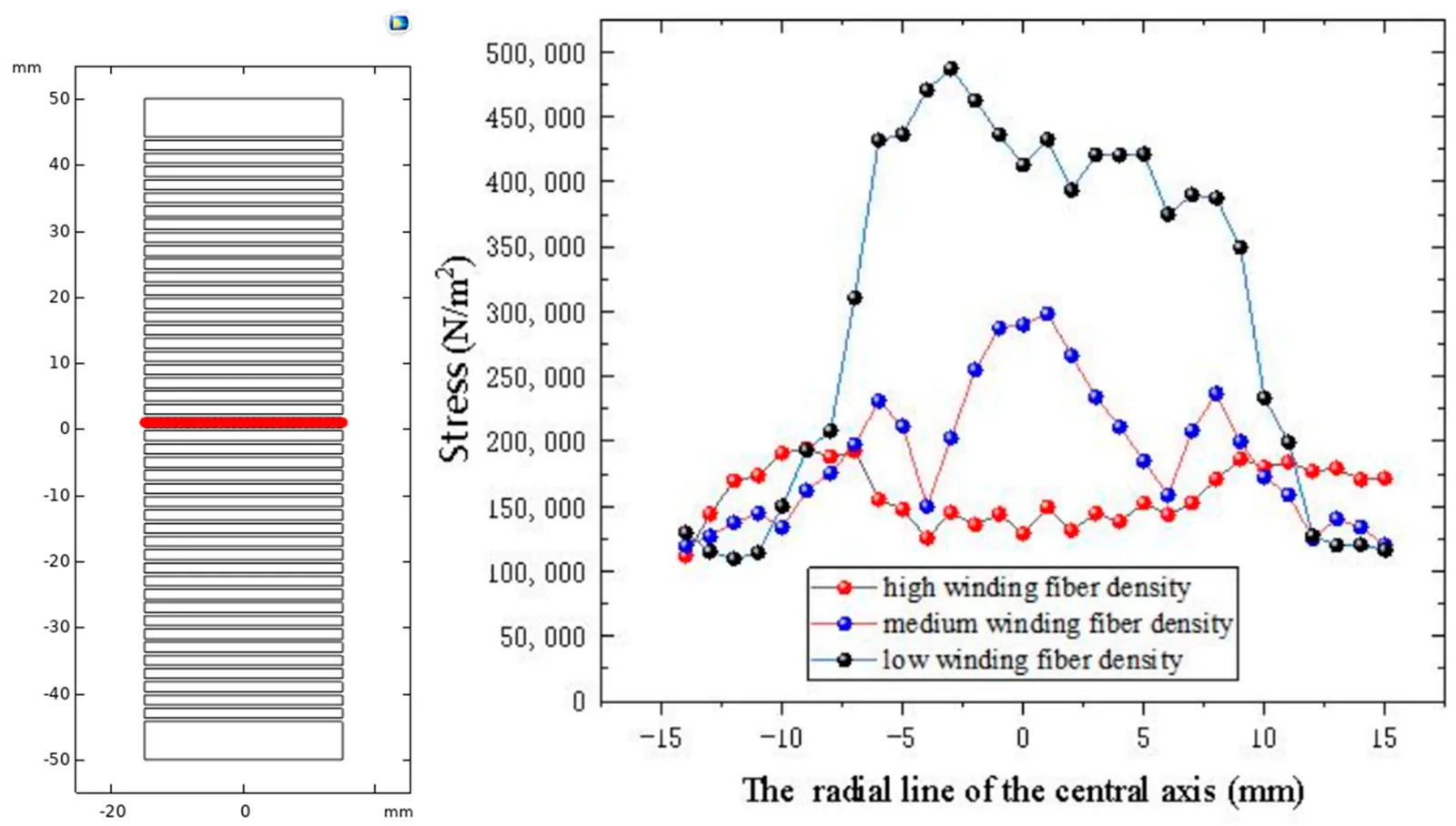
The stress curve on the radial line of the central axis of the strain layer with different winding fiber densities.

At high fiber density, the radial stress along the central axis of the strain layer varied little (Figure 12). As density decreased, the radial stress profile became wavy—low at both ends and high in the middle. Simultaneously, the surface stress concentrated along the central axis, forming a bulge.

Winding fiber density plays a critical role in determining the bending response of the soft actuator. At higher fiber densities, the stress field over both the strain and limiting layers becomes progressively more uniform. Non-uniform stress with large local gradients destabilizes the strain-layer deformation and degrades the bending response.

### 4.3. Influence of Air Cavity Offset Distance on Actuator Deformation

**Figure 13:**
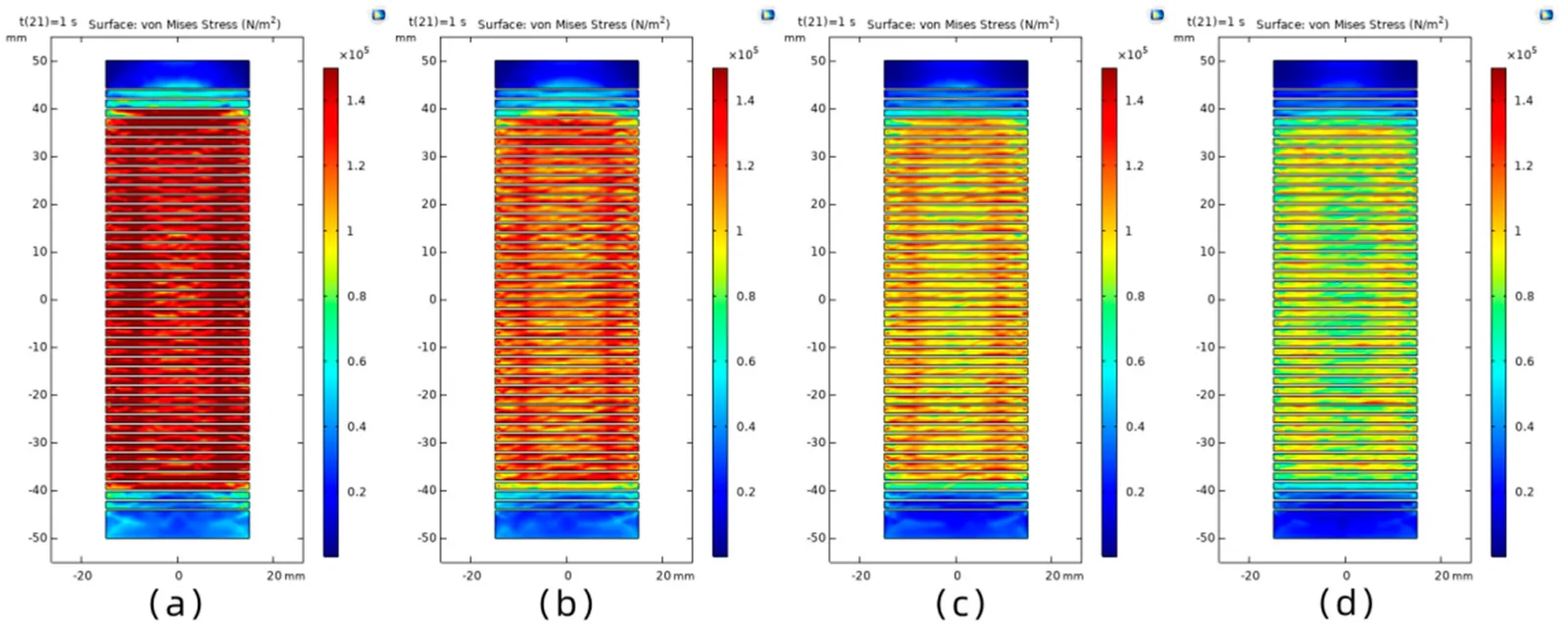
Strain-layer stress at four cavity offset distances: (**a**) 4; (**b**) 3; (**c**) 2; and (**d**) 1 mm.

As the cavity offset decreases, two effects appear (Figure 13): the strain-layer stress drops, while the limiting-layer stress rises and begins to constrain the actuator’s deformation.

**Figure 14:**
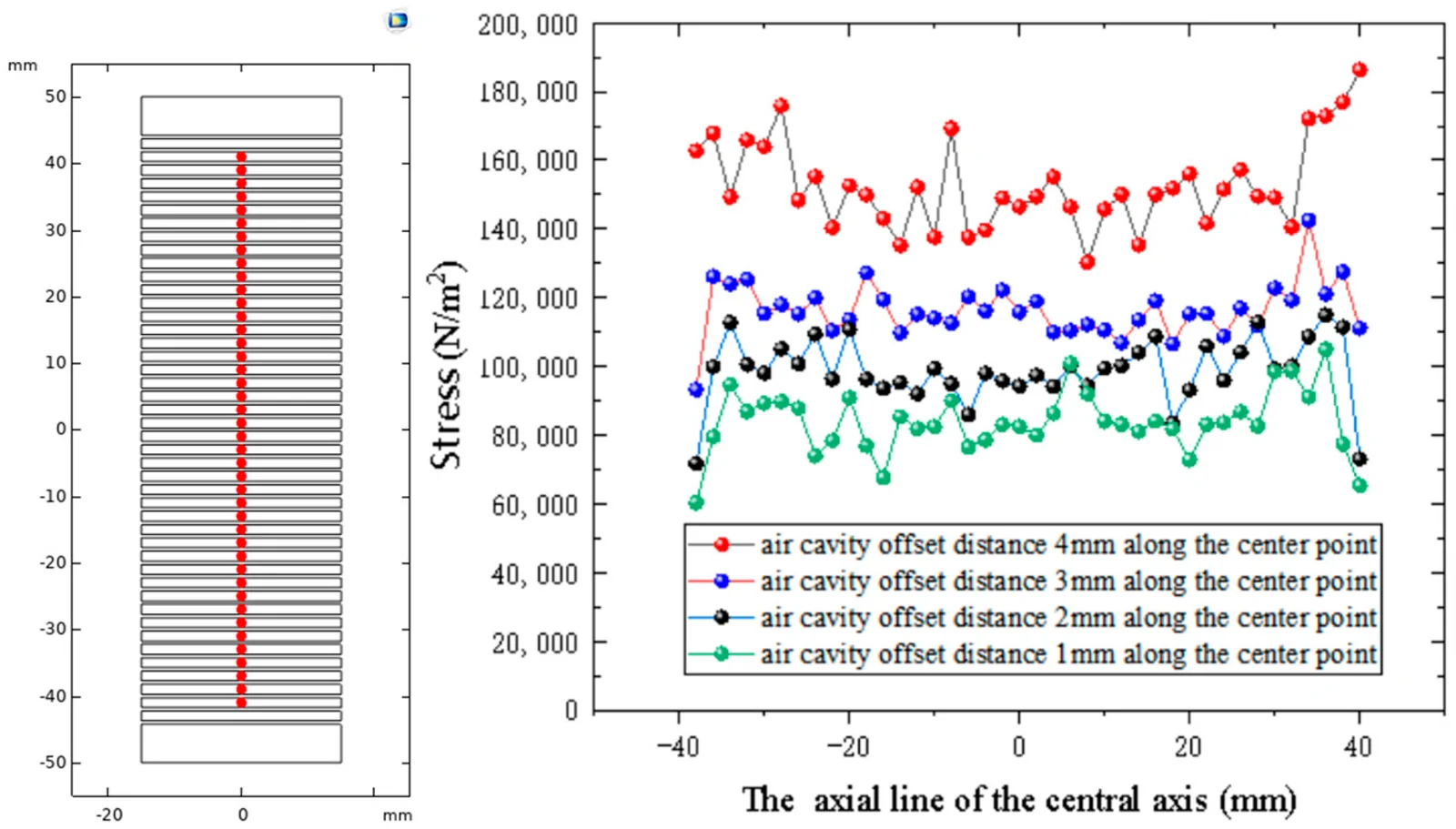
Stress variation along the axial line of the strain-layer central axis is plotted for different air cavity offset distances from the actuator center point.

The cavity offset distance significantly influenced the stress along the central axis: both the mean stress and its fluctuation amplitude increased with offset distance (Figure 14).

**Figure 15:**
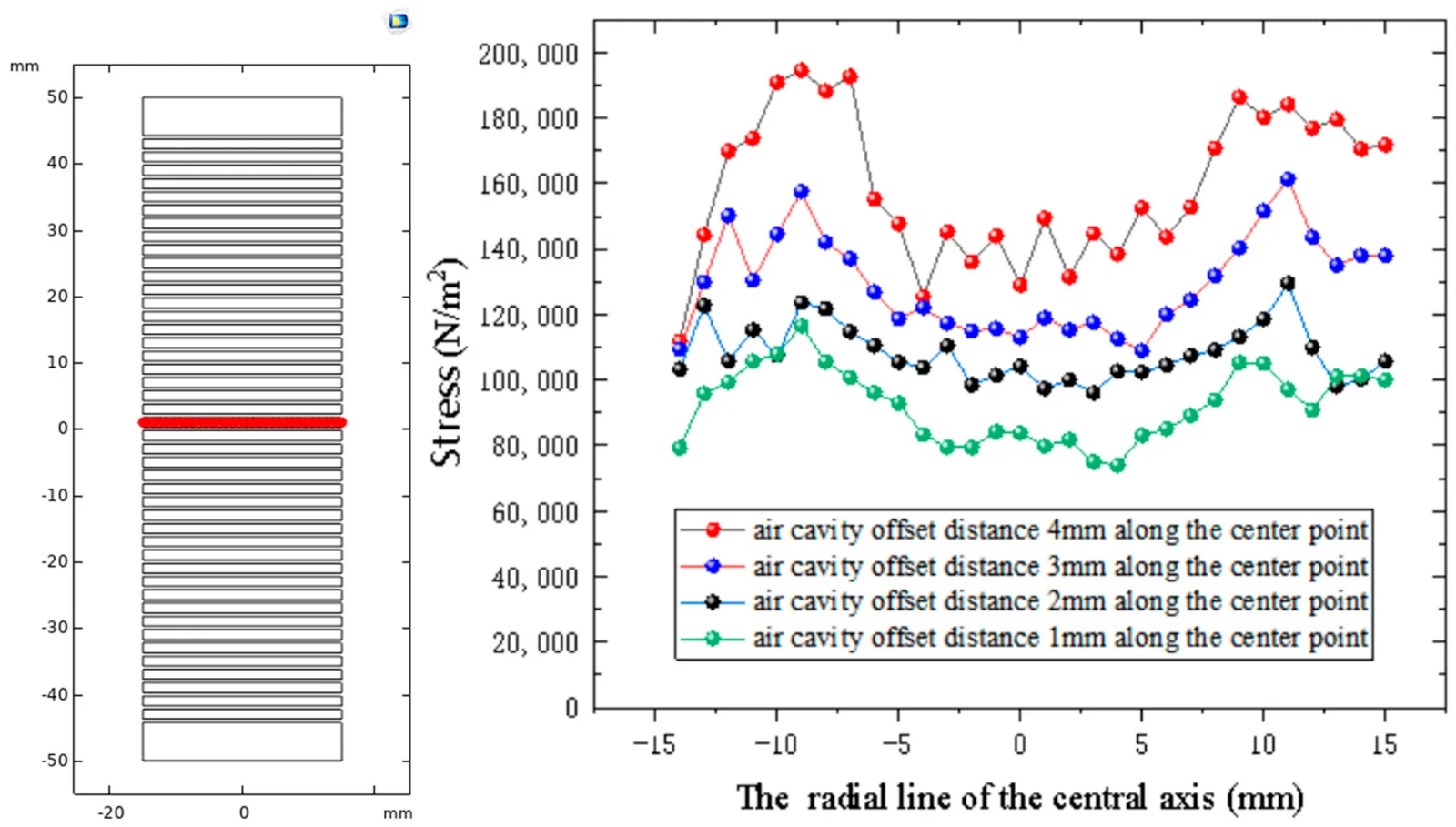
Stress distribution along the radial direction of the strain-layer central axis is presented for various air cavity offset distances from the actuator center point.

At 10 mm and −10 mm, the radial stress along the central axis peaked at the air cavity edges, where stress concentration occurred (Figure 15). In the next section, we shall analyze the stress in the air cavity.

**Figure 16:**
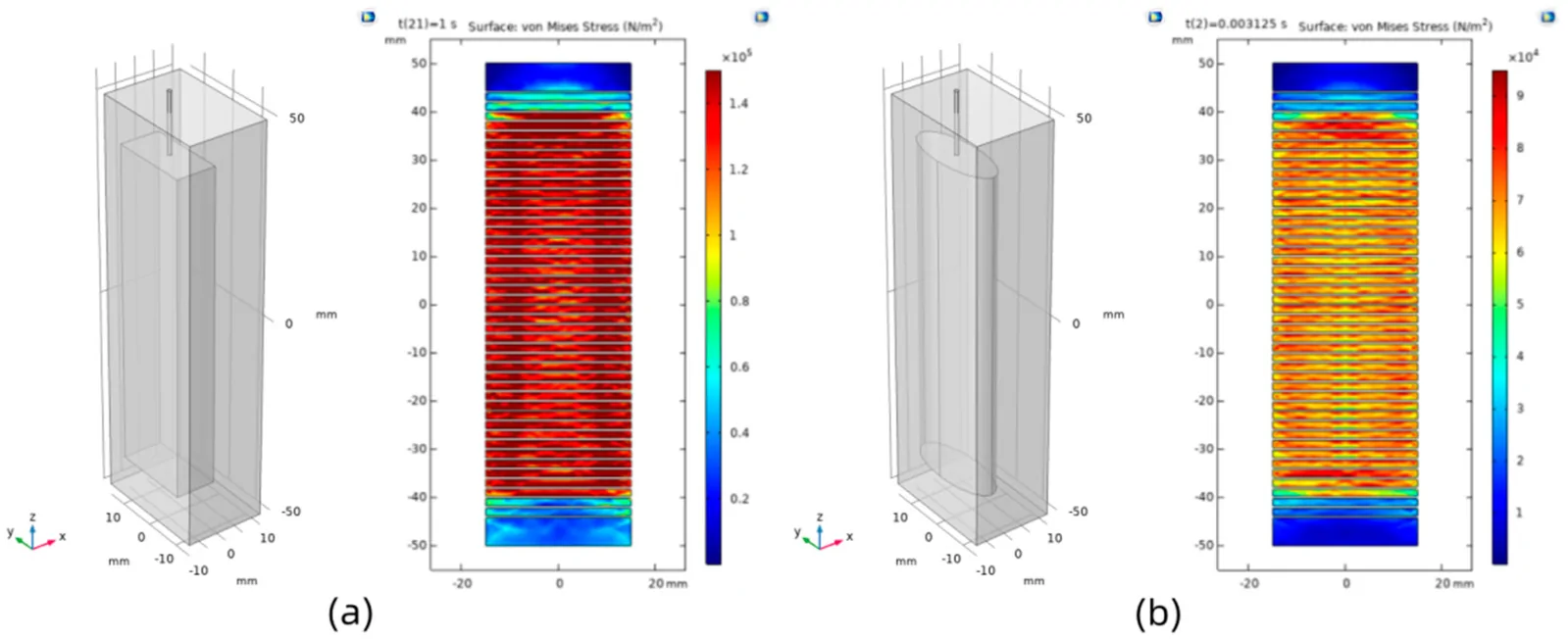
Influence of air cavity geometry on the strain-layer stress distribution: (**a**) cuboid cavity; (**b**) elliptical cylindrical cavity.

The bending deformation of the soft actuator is governed, to a large extent, by the offset distance of the air cavity from the center point. The stress rise on both the strain and limiting layers with increasing offset confirms a direct coupling between cavity eccentricity and actuation resistance (Figure 16). As the offset increases, the stress levels on both the outer and inner layers of the cavity increase proportionally, suggesting a direct relationship between eccentricity and resistance to actuation.

### 4.4. Influence of Air Cavity Geometry on Actuator Deformation

As shown in Figure 16, we chose the same volume and offset distance of the cuboid air cavity and the elliptical cylinder air cavity for comparison. The cuboid cavity produced higher average strain-layer stress, whereas the elliptical cylinder cavity yielded a more uniform stress distribution.

**Figure 17:**
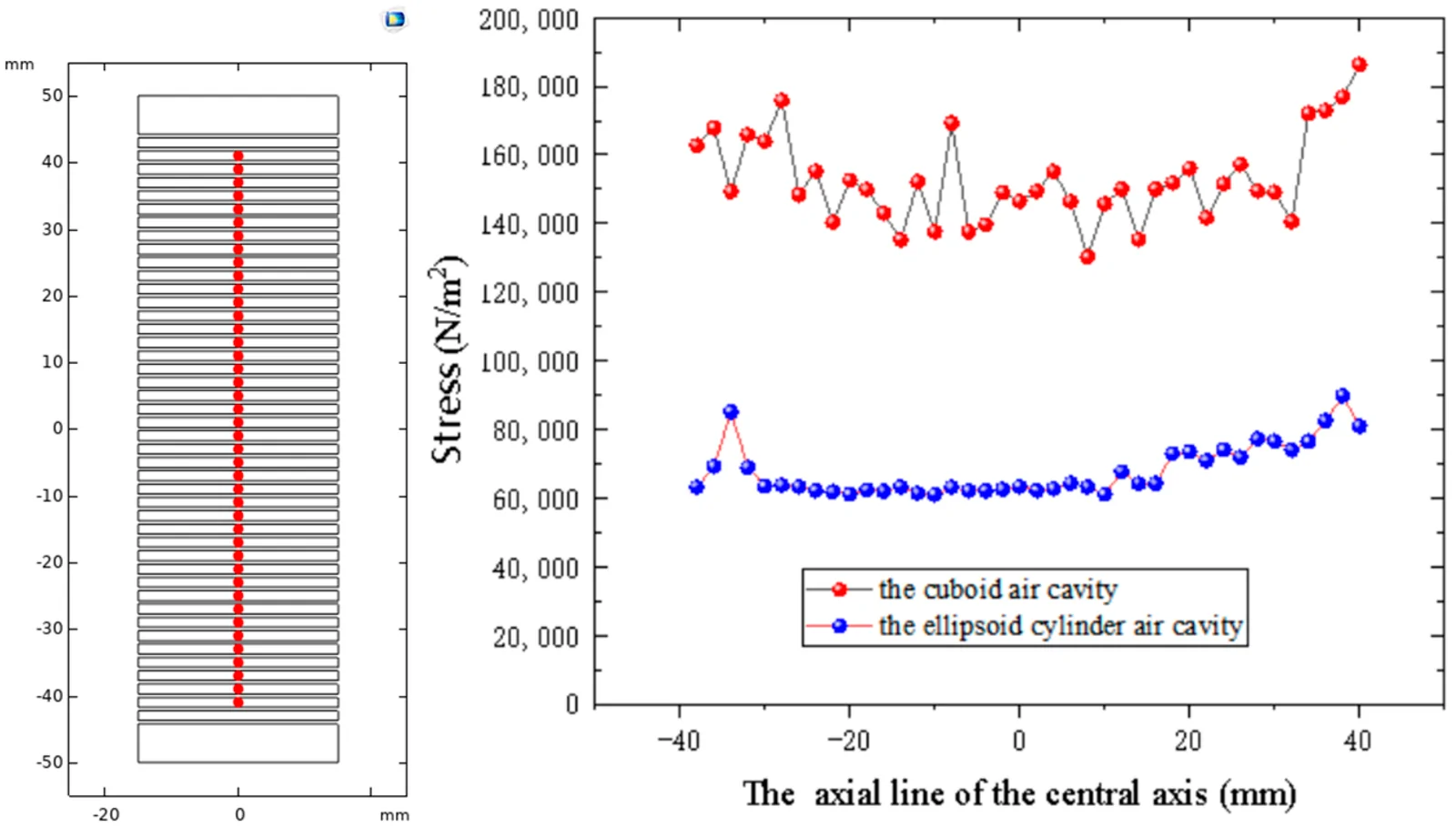
The stress curve on the axial line of the central axis of the strain layer with different air cavity structures of actuator.

The elliptical cavity produced a narrower stress range along both the axial and radial directions (Figure 17).

**Figure 18:**
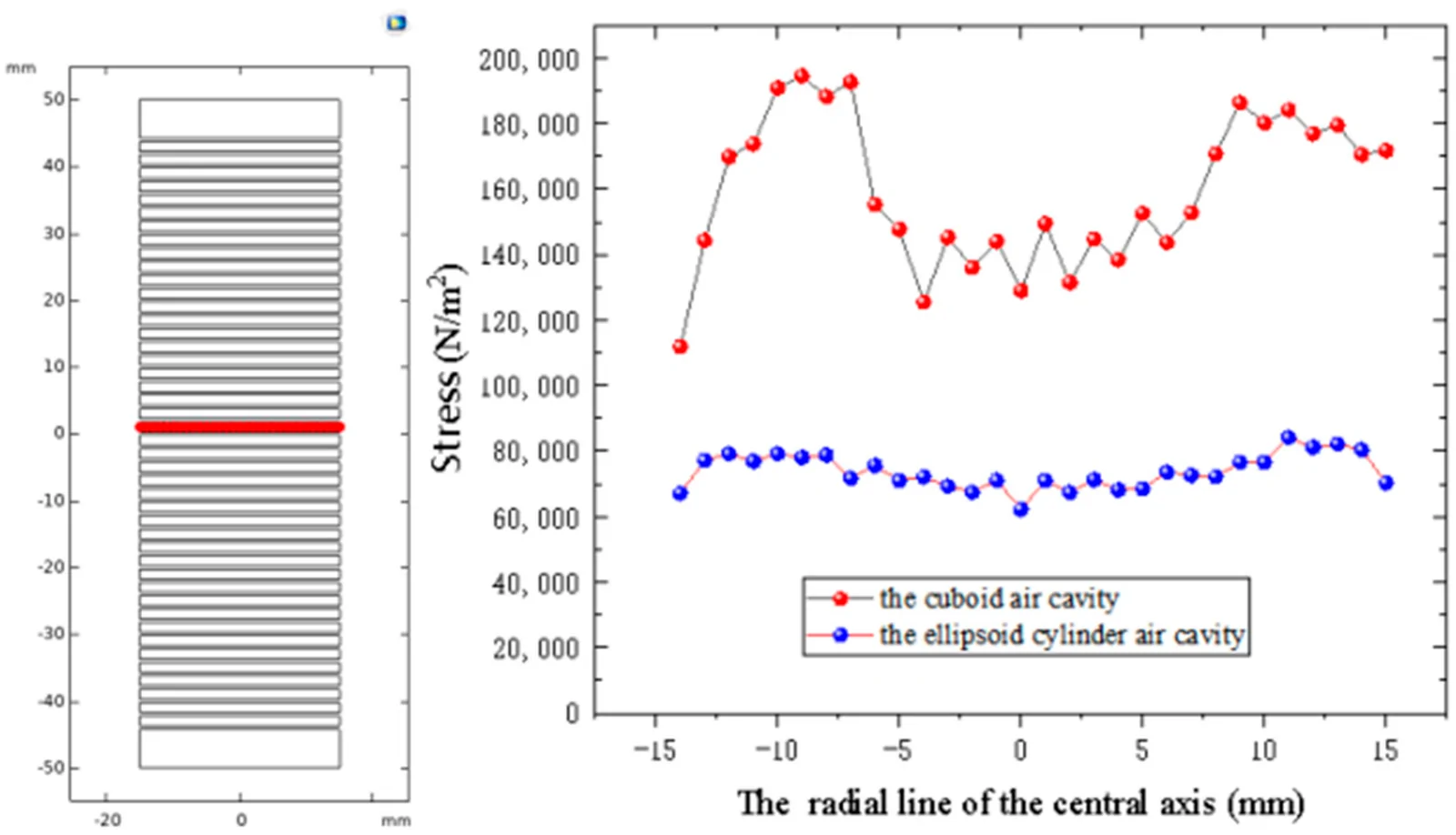
The stress curve on the radial line of the central axis of the strain layer with different air cavity structures of actuator.

The cuboid cavity produced pronounced stress fluctuations along the axial direction (Figure 18). The actuator had a larger stress at 10 mm and −10 mm on the radial line of the central axis. We studied the stress distribution in an air cavity.

**Figure 19:**
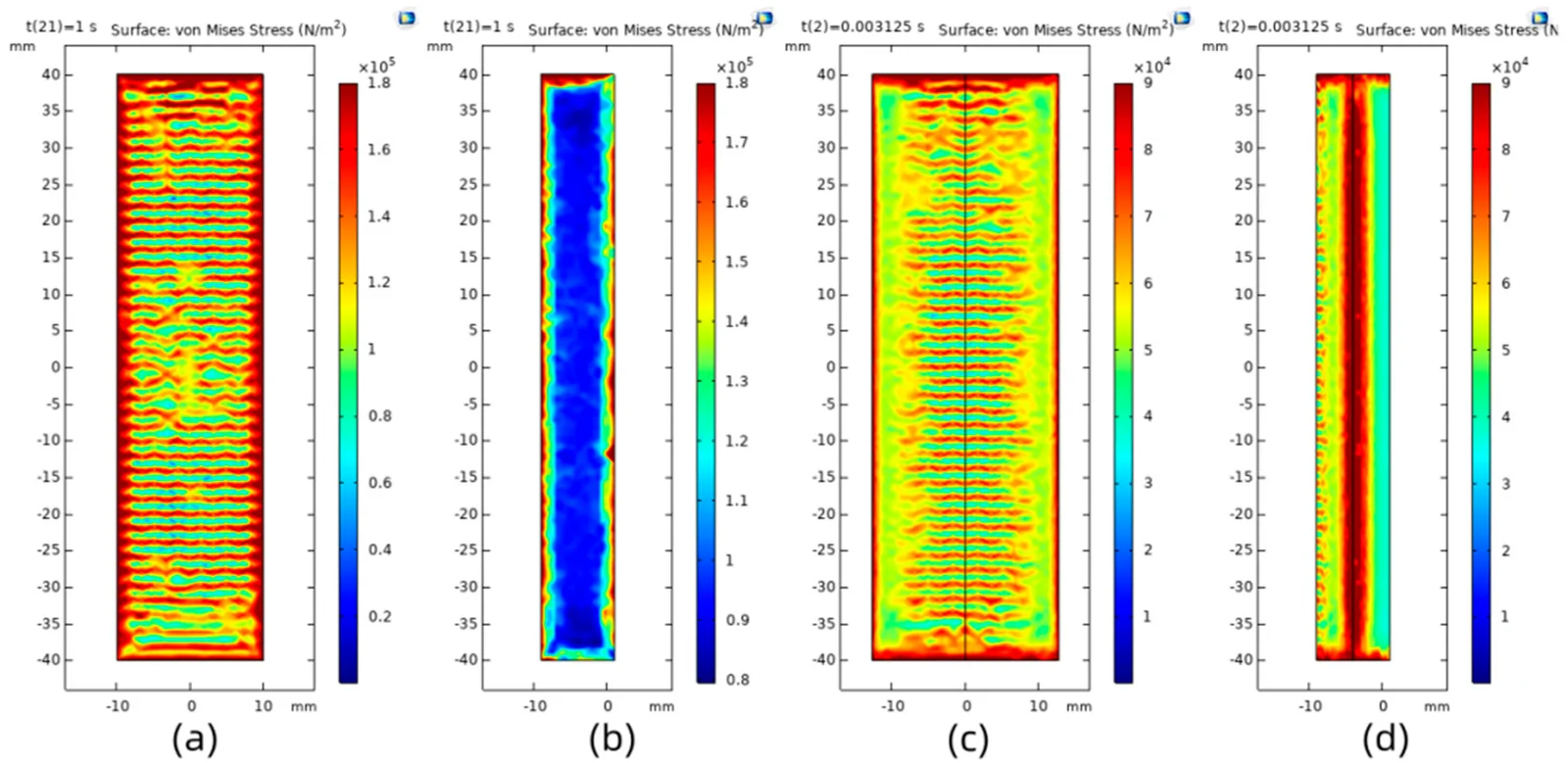
The surface (**a**) and side (**b**) stress distributions of the soft actuator cuboid air cavity, the surface (**c**) and side (**d**) stress distributions of the soft actuator ellipsoid cylinder air cavity.

Stress is concentrated at the fibers and right-angled edges of the cuboid cavity, with higher values at both corners than elsewhere and forming twin peaks at the cavity ends along the radial direction (Figure 19).

Stress concentrations also exist on the central axis at both ends of the elliptical cylinder air cavity. As shown by the stress distribution on the side of the air cavity, the stress concentration part of the elliptical cylindrical air cavity is farther from the actuator strain layer than that of the cuboid air cavity. The arc-shaped surface of the elliptical cylindrical air cavity can reduce the stress concentration and make the stress distribution of the actuator strain layer more uniform.

The stress distribution on the strain layer of the soft actuator with an elliptical cylindrical air cavity was more uniform. However, for the same air cavity volume and offset distance, the strain layer stress of the cuboid air cavity was larger. Combined with the actual actuation effect, the cuboid air cavity actuator had a larger bending angle and higher deformation efficiency.

## 5. Conclusions

We have presented a hybrid analytical–numerical framework for fiber-reinforced pneumatic soft actuators, combining a Neo-Hookean constitutive model for the isotropic silicone rubber matrix with a strain-energy formulation for the anisotropic wound fibers. The model captures the coupled extension, expansion, twist, and bending modes of the actuator and was implemented in a finite element environment to systematically investigate three key design parameters: winding fiber density, air cavity offset distance, and cavity cross-sectional geometry.

The simulations yielded three principal findings. First, higher winding fiber density promotes a uniform stress distribution across both the strain and limiting layers, whereas low fiber density induces localized bulging, large stress gradients, and degraded bending performance. Second, the offset distance of the air cavity from the neutral axis is directly coupled to the bending curvature—a larger offset produces greater cavity deformation, higher actuation efficiency, and increased stress on both layers. Third, the cuboid cavity delivers a larger bending angle and higher deformation efficiency, while the elliptical cylinder cavity distributes stress more evenly and reduces edge concentration. The simulated bending-angle response was validated against experimental measurements, with the actuator reaching a 90° bending angle at 0.045 MPa; the predicted stress-distribution trends, by contrast, are numerical results that were not directly measured in the present study.

These results provide quantitative guidance for optimizing the structure of fiber-reinforced soft actuators and demonstrate that the proposed framework can serve as a predictive tool that is independent of any specific simulation software. The parametric trends identified here—particularly the trade-off between bending efficiency and stress uniformity—offer a rational basis for selecting design parameters according to application-specific requirements.

A limitation of the current model is its reliance on a single hyperelastic formulation (Neo-Hookean), which may underestimate stiffness at large strains. In addition, the pressure is applied as a boundary load rather than through a fully coupled fluid–structure interaction framework. Future work should incorporate more accurate constitutive models, such as the Ogden or Yeoh forms [29], as well as a full FSI formulation with ALE moving-mesh treatment, compressible airflow modeling (including an appropriate equation of state, inlet/outlet conditions, and dynamic pressure response), and transient analysis capabilities. Extending the present framework to dynamic loading conditions and multi-chamber actuator geometries also represents an important direction for further investigation.

It should be noted that the present experimental validation was limited to the bending angle—a global deformation metric measured from photographic images. The internal stress distribution predicted by the finite element model has not been directly validated, owing to the difficulty of embedding stress sensors in soft hyperelastic materials without disturbing their mechanical behavior. In future work, full-field strain measurement techniques such as digital image correlation (DIC) could be employed to experimentally capture the surface strain fields of the actuator under pressurization, enabling a more comprehensive validation of the simulated stress and strain distributions. Such validation would further strengthen the predictive capability of the proposed hybrid analytical–numerical framework.

## Data Availability

The original contributions presented in this study are included in the article. Further inquiries can be directed to the corresponding author.
